# Targeting p300 and CBP abolishes HOXB13-loss-induced lipogenesis and tumor metastasis

**DOI:** 10.1172/jci.insight.195743

**Published:** 2025-11-24

**Authors:** Xiaodong Lu, Liu Peng, Qi Chu, Samantha Ye, Mingyang Liu, Maha Hussain, Mehmet A. Bilen, Lara R. Harik, Jonathan Melamed, Jonathan C. Zhao, Jindan Yu

**Affiliations:** 1 Department of Urology, Emory University School of Medicine, Atlanta, Georgia, USA.; 2 Division of Hematology/Oncology, Department of Medicine, Northwestern University Feinberg School of Medicine, Chicago, Illinois, USA.; 3 Department of Hematology and Medical Oncology, Emory University School of Medicine, Atlanta, Georgia, USA.; 4 Winship Cancer Institute, Emory University School of Medicine, Atlanta, Georgia, USA.; 5 Department of Pathology and Laboratory Medicine, Emory University School of Medicine, Atlanta, Georgia, USA.; 6 Department of Pathology at NYU Grossman School of Medicine, New York, New York, USA.; 7 Department of Human Genetics, Emory University School of Medicine, Atlanta, Georgia, USA.

**Keywords:** Genetics, Oncology, Drug therapy, Epigenetics, Prostate cancer

## Abstract

HOXB13 is a prostate-specific transcription factor best known for its role as an androgen receptor (AR) cofactor. Recent evidence suggests that HOXB13 plays critical AR-independent functions in repressing lipogenic programs and promoting prostate cancer (PCa) metastasis. However, the mechanisms linking HOXB13 loss to tumor metastasis remain unclear. Here, we show that p300 and CBP co-occupy lipogenic enhancers suppressed by HOXB13 and HDAC3 and are essential for enhancer activation and target gene expression following HOXB13 depletion. Loss of HOXB13 induces lipid-sensitive matrix metalloproteinases (MMPs), promoting increased cell motility. Importantly, pharmacological inhibition of p300 and CBP blocks HOXB13-loss-driven lipogenesis, reduces MMP expression, and decreases cell migration in vitro and tumor metastasis in vivo. Analysis of clinical samples revealed that HOXB13 expression is reduced in metastatic hormone-sensitive PCa compared with matched primary tumors, further supporting its role in tumor metastasis. These findings demonstrate that HOXB13 downregulation promotes PCa metastasis through p300- and CBP-dependent lipogenic and motility pathways, which may be targeted by p300 inhibition.

## Abstract 2

HOXB13 loss promotes metastasis in prostate cancer via p300/CBP-driven lipogenesis and MMP induction, revealing a therapeutic vulnerability to p300/CBP inhibition.

## Graphical abstract

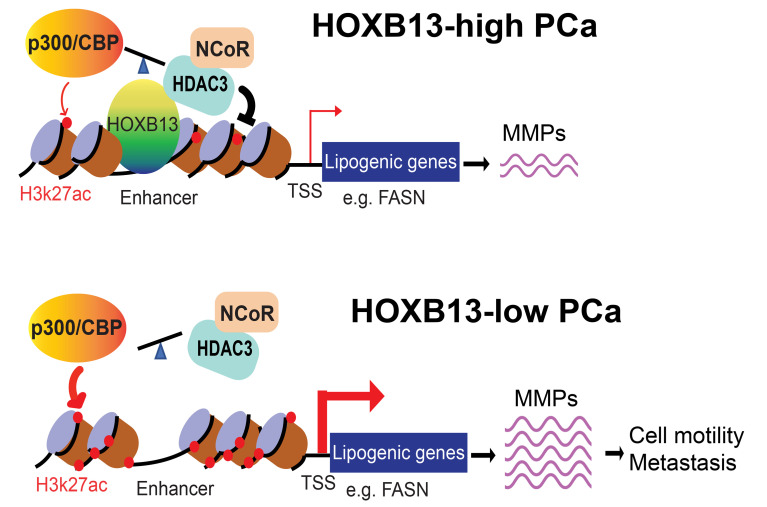

## Introduction

HOXB13, a member of the homeobox (HOX) family of transcription factors, is predominantly expressed in the adult prostate and plays a critical role in regulating prostate tissue–specific gene expression (1–4). Transgenic mouse studies have shown that HOXB13, known as a pioneering cofactor of the androgen receptor (AR), is required for the normal differentiation and secretory function of the prostate (5). Dysregulation of HOXB13 promotes prostate cancer (PCa) through diverse mechanisms (6–8). In androgen-dependent PCa, HOXB13 modulates AR activity by facilitating, recruiting, or repressing AR binding at specific *cis*-regulatory elements across the genome (6). In castration-resistant PCa (CRPC), HOXB13 mainly colocalizes with AR-V7, a CRPC-associated AR variant, to govern AR-V7–driven oncogenic programs, including cell proliferation (8). Our recent study reported an AR-independent function of HOXB13 (9). We found that HOXB13 interacts with the histone deacetyltransferase 3 (HDAC3)–nuclear receptor co-repressor (NCoR) complex, and recruits them to lipogenic enhancers to catalyze deacetylation of histone H3 at lysine 27 (H3K27ac), leading to the inhibition of lipogenic programs, independently of AR (9). Accordingly, the loss of HOXB13, observed in approximately 30% of metastatic CRPC tumors, activates a lipogenic transcriptional program.

The histone acetyltransferases CREBBP (CBP) and EP300 (p300) are key enzymes that catalyze H3K27ac, activating enhancers, facilitating transcriptional machinery recruitment, and promoting promoter-enhancer looping (10–12). CBP and p300 function antagonistically to the HDAC3-NCoR co-repressor complex in regulating steady-state H3K27ac dynamics and fine-tuning individual enhancer activities (10, 13, 14). Both p300 and CBP are highly expressed in advanced PCa, and their elevated expression correlates with poor clinical outcomes (15, 16). Additionally, recent evidence indicates that p300 transcriptionally upregulates *FASN*, leading to increased lipid accumulation in PCa cells (17). However, it remains unexplored whether p300 and CBP are involved in mediating HOXB13-loss-induced lipogenic programs in PCa cells.

Accumulation of lipid droplets has been seen in high-grade, metastatic PCa and circulating prostate tumor cells (18). Changes in lipid metabolism have been shown to significantly contribute to PCa progression and metastasis (19, 20). AR is a major regulator of lipid metabolic genes involved in lipid synthesis, uptake, and storage (21, 22), and in CRPC, reactivation of AR-induced lipid biosynthesis has been linked to tumor growth and metastasis (23). In addition, genetic perturbation of tumor suppressor genes *Pten* and *Pml*, or overexpression of subunits of the pyruvate dehydrogenase complex (PDC), such as *Pdhda1*, aberrantly activate sterol regulatory element–binding protein–dependent (SREBP-dependent) lipogenic programs, leading to mouse prostate tumor growth and metastasis (24–26). Moreover, a high-fat diet (HFD) has been shown to enhance PCa progression (27). We recently showed that HOXB13 loss increases lipid accumulation, thereby promoting PCa cell motility in vitro and PCa metastasis in vivo (9). However, the signaling pathways by which HOXB13-loss-induced lipogenesis promote PCa metastasis remain elusive.

Here, we found that HOXB13 is downregulated in metastatic PCa compared with its primary counterparts. HOXB13 loss disrupts HDAC3 and p300/CBP equilibrium at lipogenic enhancers, leading to increased p300 and CBP recruitment and H3K27ac, thereby aberrantly activating lipogenic programs. HOXB13-loss-induced lipids promote PCa cell motility through upregulation of matrix metalloproteinases (MMPs). Such effects of HOXB13 loss are effectively blocked by knockdown (KD) or pharmacological inhibition of p300 and CBP proteins.

## Results

### p300 co-occupies and activates HOXB13/HDAC3-bound lipogenic enhancers.

**Figure 1:**
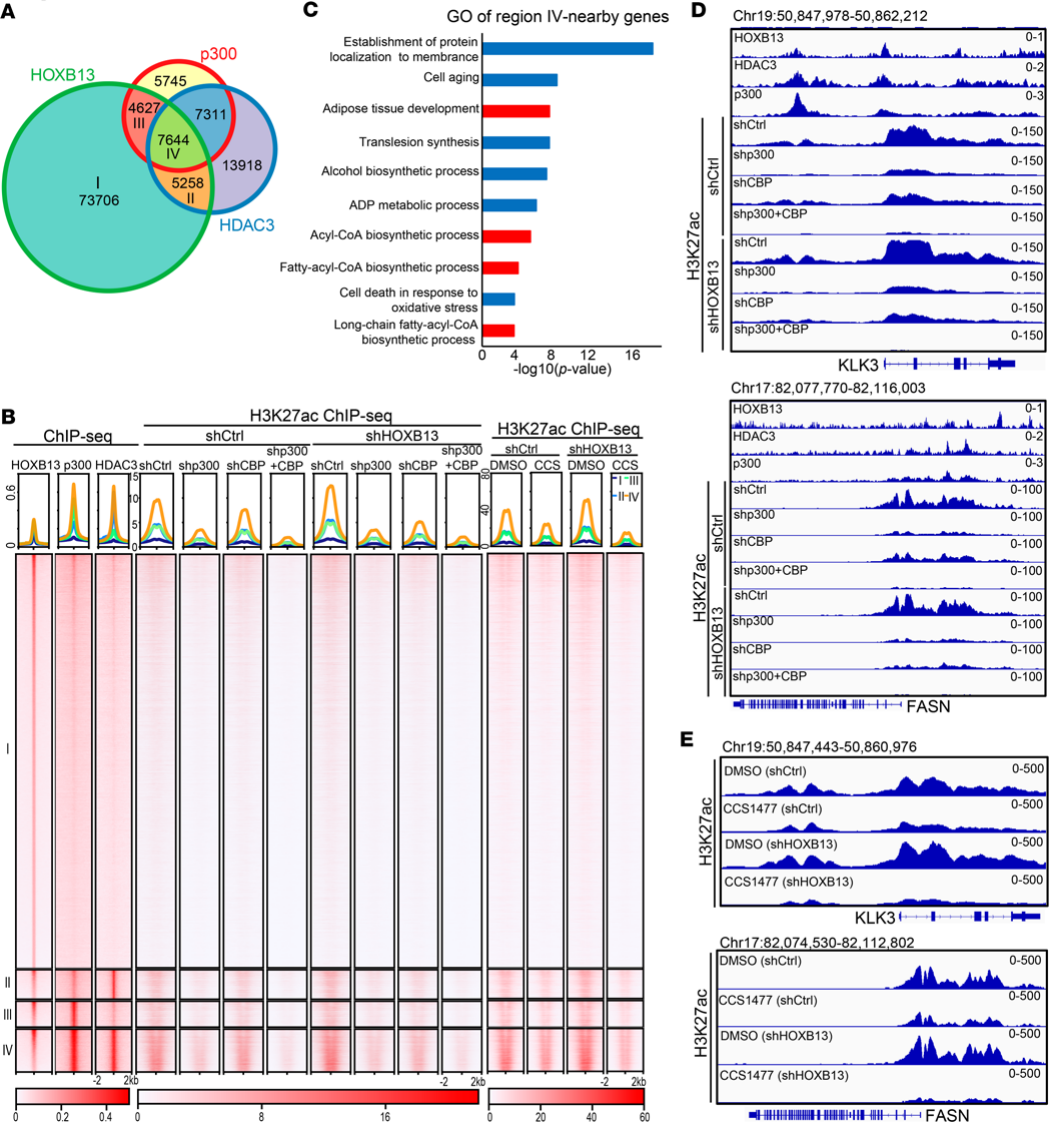
p300 co-occupies and activates HOXB13/HDAC3-bound lipogenic enhancers. (**A**) Venn diagram showing overlap of HOXB13, p300, and HDAC3 binding sites in LNCaP cells. (**B**) Heatmaps showing indicated ChIP-seq intensity centered (±2 kb) around the 4 clusters of overlapping sites identified in **A**. I, HOXB13-only sites; II, HOXB13 and HDAC3 co-binding sites; III, HOXB13 and p300 co-binding sites; IV, HOXB13, HDAC3, and p300 co-binding sites (HHP). Scale bar: enrichment intensity. (**C**) GO analysis genes near the HOXB13, HDAC3, and p300 co-binding site (region IV in **A**), identified by GREAT, with gene regulatory domain definition within 100 kb. *P* values by binomial test. (**D**) IGV showing HOXB13, p300, HDAC3, and H3K27ac ChIP-seq signal at HOXB13-repressed genes *KLK3* (top) and *FASN* (bottom) loci in LNCaP cells with indicated genetic aberrations. (**E**) IGV showing H3K27ac ChIP-seq signal at HOXB13-repressed genes *KLK3* (top) and *FASN* (bottom) loci in control or *HOXB13*-KD LNCaP cells with DMSO or CCS1477 treatment.

Our recent study demonstrated that HOXB13 suppresses lipogenic gene expression via recruitment of the HDAC3-NCoR corepressor complex, whereas HOXB13 loss activates the lipogenic programs (9). Given that p300 and CBP are principal antagonists of HDAC3 and NCoR in catalyzing H3K27ac (10, 13), we hypothesized that HOXB13 loss activates lipogenic genes by requiring p300 and CBP. To test this, we performed chromatin immunoprecipitation followed by next-generation sequencing (ChIP-seq) for HDAC3 and p300 in LNCaP cells and compared their binding sites with the HOXB13 cistrome. We observed significant co-occupancy of HDAC3 and p300 at some HOXB13 binding sites (Figure 1A, region IV). Notably, these co-bound sites exhibited markedly higher binding of both HDAC3 and p300, as well as stronger H3K27ac signal, compared with regions bound by HDAC3 or p300 alone (Figure 1B, left 3 lanes). These findings are consistent with previous studies showing that HDACs and histone acetyltransferases (HATs) often colocalize at transcriptionally active genes to fine-tune gene expression (13). To gain some insights into the function of peak-associated genes, we performed Genomic Regions Enrichment of Annotations Tool (GREAT) analysis (28) to identify nearby genes, which revealed 45 genes, including *FASN*, that are involved in the fatty-acyl-CoA biosynthetic process and long-chain fatty-acyl-CoA biosynthetic process (Figure 1C and Supplemental Table 1; supplemental material available online with this article; https://doi.org/10.1172/jci.insight.195743DS1). These findings are in agreement with our previous reports of HOXB13 and HDAC3 in regulating lipid metabolism (9) and further suggest that HAT proteins are also involved.

To determine whether p300 and CBP are required for H3K27ac at lipogenic enhancers following HOXB13 loss, we performed H3K27ac in *HOXB13*-KD cells with concurrent CBP and/or p300 KD (Supplemental Figure 1A). As expected, H3K27ac levels were increased upon *HOXB13* KD (Figure 1B). Significantly, these HOXB13-loss-induced increases in H3K27ac were substantially attenuated by *p300* KD, and to a lesser extent by *CBP* KD (Figure 1B), indicating that p300 plays a predominant role in enhancer activation following HOXB13 loss. This was further supported by Integrative Genomics Viewer (IGV) tracks, which showed increased H3K27ac at known HOXB13-repressed genes, including *KLK3* and *FASN*, upon *HOXB13* KD, which was reversed by *p300* or *CBP* KD (Figure 1D). To further validate this finding, we performed H3K27ac ChIP-seq in control and *HOXB13*-KD LNCaP cells treated with either DMSO or CCS1477, a selective p300/CBP bromodomain inhibitor currently in clinical trials for advanced PCa and hematologic malignancies (16, 29). As observed with genetic depletion, pharmacologic inhibition with CCS1477 dramatically reduced *HOXB13*-KD-induced H3K27ac globally (Figure 1B, right 4 lanes) as well as at representative sites, such as *KLK3* and *FASN* enhancers (Figure 1E), which was further confirmed by ChIP-qPCR (Supplemental Figure 1B). Collectively, these data demonstrate that HOXB13 loss disrupts the balance between HDAC3 and p300 at lipogenic enhancers, leading to increased H3K27 acetylation that is abolished by CBP and p300 inhibition.

### p300 and CBP are required for HOXB13-loss-induced lipogenic program.

**Figure 2:**
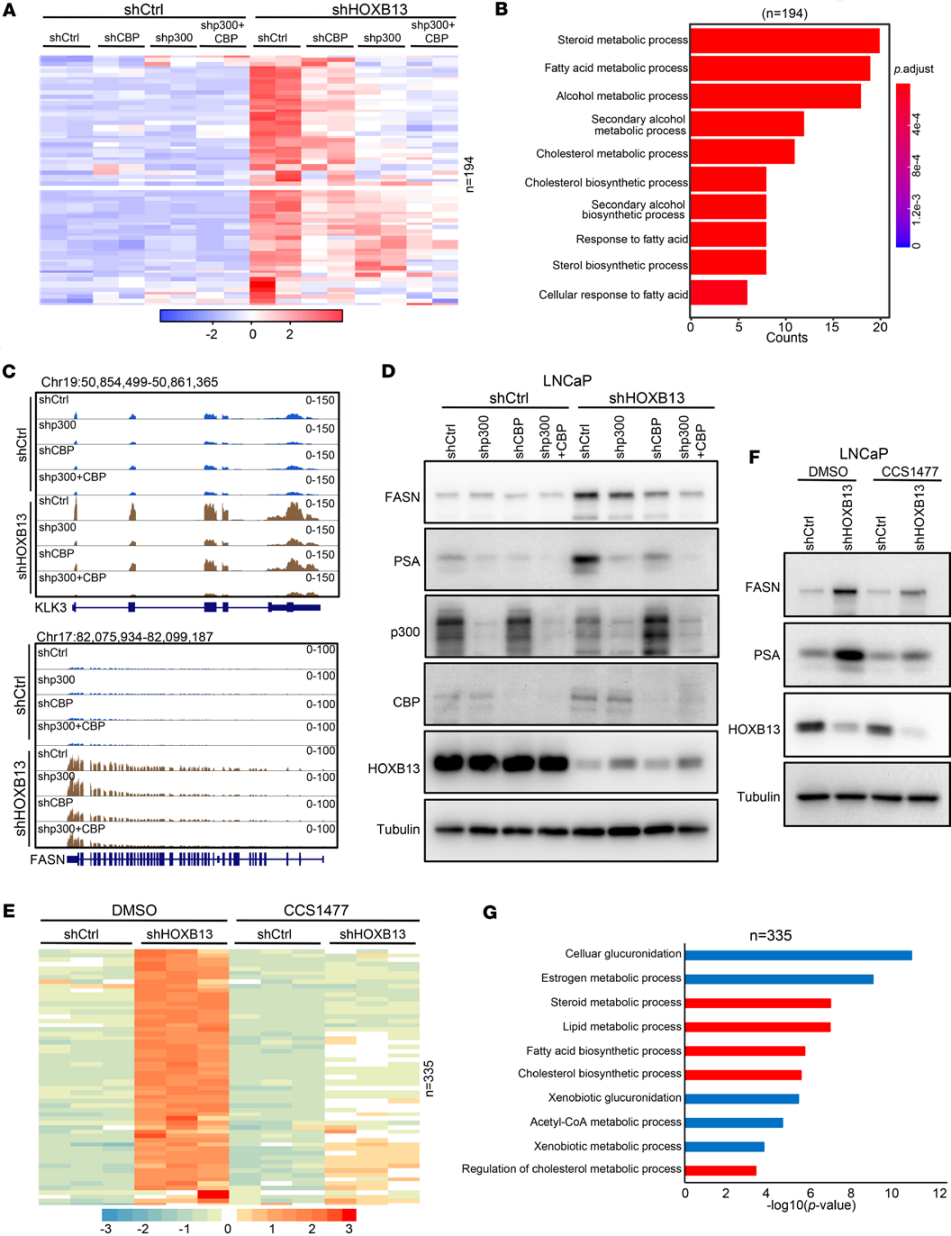
p300 and CBP are required for HOXB13-loss-induced lipogenic program. (**A**) Heatmap showing shHOXB13-induced genes that are dependent on p300 and CBP in LNCaP cells with *HOXB13* KD and/or *p300*/*CBP* KD. HOXB13-repressed genes were identified by DESeq2 with fold change ≥ 1.5 and adjusted *P* < 0.05. Color bar: *z*-score. (**B**) GO analysis of the genes identified in **A**. Top enriched molecular concepts are shown on the *y*-axis, while the *x*-axis indicates number of genes in each significant GO term. (**C**) Genome browser showing mRNA expression of HOXB13-target genes *KLK3* and *FASN* in LNCaP cells with indicated treatment. (**D**) Western blot analysis of FASN, PSA, and HOXB13 expression in LNCaP cells with *HOXB13* KD and/or *p300*/*CBP* KD. (**E**) Heatmap showing shHOXB13-induced genes that are repressed by CCS1477 in LNCaP cells with *HOXB13* KD and/or CCS1477 treatment. Control (sh*Ctrl*) or *HOXB13*-KD (sh*HOXB13*) cells were subjected to control (DMSO) or CCS1477 (250 nM) treatment for 48 hours, and RNA-seq was performed in triplicate. HOXB13-repressed genes were identified by DESeq2 with fold change ≥ 2 and adjusted *P* < 0.05. Color bar: *z*-score. (**F**) Western blot analysis of FASN, PSA, and HOXB13 expression in control or *HOXB13*-KD LNCaP cells treated with DMSO or CCS1477. (**G**) GO analysis of the genes identified in **E**. Top enriched molecular concepts are shown on the *y*-axis, while the *x*-axis indicates enrichment significance. Lipid- and cell cycle–related GO terms are highlighted in Red. *P* values by 1-sided hypergeometric test.

Next, to evaluate whether HOXB13 loss induces lipogenic programs through p300/CBP, we performed RNA-seq in control and *HOXB13*-KD LNCaP cells with concomitant *p300* and/or *CBP* KD. We identified 194 genes increased by *HOXB13* KD (fold change ≥ 1.5, adjusted *P* < 0.05) that were significantly rescued upon *p300/CBP* depletion (Figure 2A). Gene Ontology (GO) analysis revealed that these genes were significantly enriched for steroid metabolism, lipid biosynthesis, and fatty acid metabolic processes (Figure 2B), suggesting that HOXB13-loss-induced lipogenic gene programs are highly dependent on p300 and CBP activity.

Taking a closer look at these 194 genes, we found that they were more sensitive to *p300* KD than *CBP* KD (Figure 2A), consistent with earlier findings showing that HOXB13-loss-induced H3K27ac enrichment was more substantially reversed by *p300* than *CBP* depletion. Importantly, in control cells, these genes were barely affected by *p300*/*CBP* KD, suggesting that the loss of HOXB13 created a dependency on p300 and CBP. These findings were validated by representative HOXB13-repressed genes, such as *KLK3* and *FASN*, that were increased upon *HOXB13* KD, which was significantly attenuated by *p300* KD, and to a lesser extent by *CBP* KD (Figure 2, C and D).

To further examine whether these HOXB13-loss-induced downstream genes can be targeted using pharmacological inhibitors, we performed RNA-seq in control and *HOXB13*-KD LNCaP cells treated with DMSO or 250 nM CCS1477 for 48 hours. Integrative analysis of shHOXB13-induced and CCS1477-repressed genes identified 335 genes that were markedly upregulated upon *HOXB13* KD and significantly downregulated by CCS1477 (Figure 2E), consistent with our genetic perturbation results. Likewise, these genes were barely affected by CCS1477 in HOXB13-expressing control cells, indicating that loss of HOXB13 creates a vulnerability to p300/CBP inhibition. Globally, shHOXB13-induced genes accounted for the majority of the genes that were strongly repressed by CCS1477 (Supplemental Figure 1C), further supporting an on-target effect. Western blot analysis of representative proteins, including PSA and FASN, confirmed this effect; their expression was elevated in *HOXB13*-KD cells and reduced upon CCS1477 treatment (Figure 2F). GO analysis revealed that these shHOXB13-induced and CCS1477-targeted genes (Figure 2E) were enriched for lipid-related pathways (Figure 2G), consistent with the genetic perturbation data described earlier, Collectively, these data demonstrate that HOXB13 loss induces a lipogenic transcriptional program that is critically dependent on p300 and CBP activity.

### HOXB13 is downregulated in metastatic hormone-sensitive PCa.

**Figure 3:**
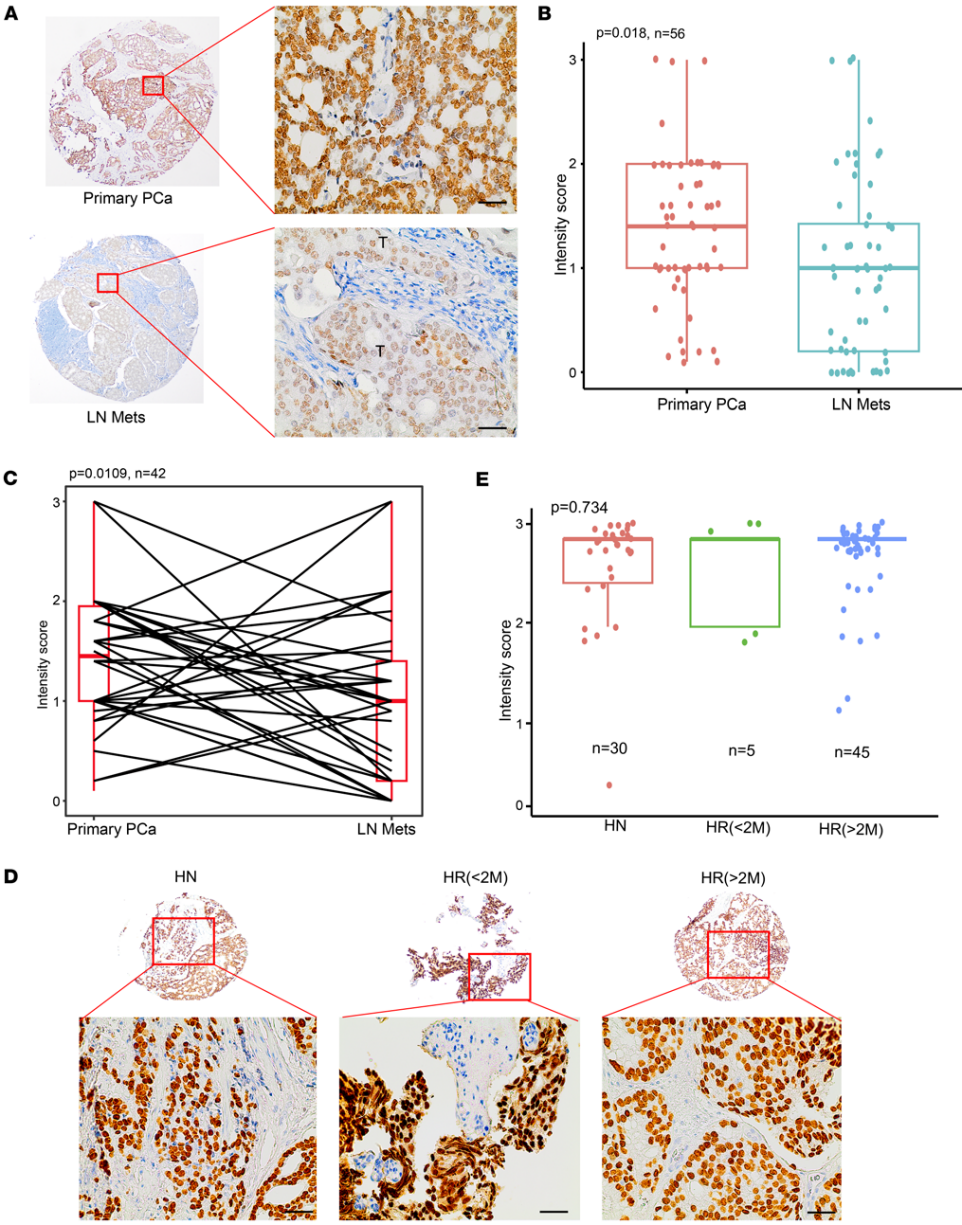
HOXB13 is downregulated in metastatic hormone-sensitive prostate cancer. (**A**) Representative images of HOXB13 staining in primary tumors (top) and lymph node–metastatic PCa (LN Mets, bottom) are shown in low (×4) and high (×40) magnification. “T” indicates tumor in LN Mets. (**B**) Quantification of HOXB13 IHC staining intensities in primary PCa and LN Mets. *P* value was calculated by unpaired, 2-sided *t* test. Box-and-whisker plots represent the median (line) and bottom and upper quartiles (box bounds); whisker edges indicate the minimum and maximum values. (**C**) Quantification of HOXB13 IHC staining intensities in paired primary PCa and LN Mets. Data are shown as in **B**. *P* value was calculated by paired, 2-sided *t* test. (**D**) Representative images of HOXB13 staining in primary PCa from hormone treatment–naive patients (hormone naive, HN; left), or from patients with hormone therapy less than 2 months (hormone received, HR<2M, middle), or from patients with hormone therapy more than 2 months (hormone received, HR>2M, right). The images are shown in low (×10, top) and high (×40, bottom) magnification. (**E**) Quantification of HOXB13 IHC staining intensities in PCa samples from **D**. Data are shown as in **B**. *P* value was calculated by 1-way ANOVA followed by Tukey’s multiple-comparison test.

We and others have recently reported that HOXB13 is downregulated in approximately 30% of metastatic CRPC and a majority of neuroendocrine PCa (9, 30, 31). Although a previous study has shown a trend of low HOXB13 being associated with worse metastasis-free survival (30), HOXB13 expression in hormone-sensitive metastatic versus primary PCa has not been investigated. To address this, we measured HOXB13 expression by immunohistochemistry (IHC) in an Emory University cohort (Emory cohort) that consisted of 56 high-grade primary PCa cases and 56 lymph node–metastatic (LN-metastatic) PCa cases, all from patients with no prior history of hormone therapy, that is, hormone-naive (HN). Among these, 42 cases had matched primary and LN-metastatic tumor samples from the same patient. IHC analysis showed that HOXB13 was primarily localized to the nucleus, consistent with its function as a transcription factor (Figure 3A). Critically, HOXB13 expression was overall significantly decreased in LN metastases compared with primary PCa (Figure 3, A and B). Taking a closer look at matched primary and LN cases, we confirmed significantly lower HOXB13 staining in the LN-metastatic lesions compared with the matched primary tumors (Figure 3C), suggesting that HOXB13 downregulation is associated with metastatic progression.

Given that HOXB13 is a key cofactor of AR, we wondered whether its expression might be influenced by androgen deprivation therapy (ADT), a standard treatment for advanced PCa. To this end, we exploited the New York University cohort (NYU cohort) of primary PCa that included 30 HN, 5 samples from patients who had received hormone therapy for less than 2 months (HR<2M), and 45 samples from patients treated for more than 2 months (HR>2M). IHC staining revealed that HOXB13 expression was not significantly altered in patients who had received ADT, regardless of treatment duration, when compared to HN samples (Figure 3, D and E). These data suggest that HOXB13 is not directly regulated by ADT, nor did it undergo upregulation during the development of treatment resistance to ADT. In summary, our results indicate that HOXB13 downregulation occurs early at the metastatic processes of PCa, rather than as a consequence of hormone therapy, thus representing a critical therapeutic target in metastatic hormone-sensitive PCa.

### Lipid-induced MMP genes mediate HOXB13-loss-induced cell motility.

**Figure 4:**
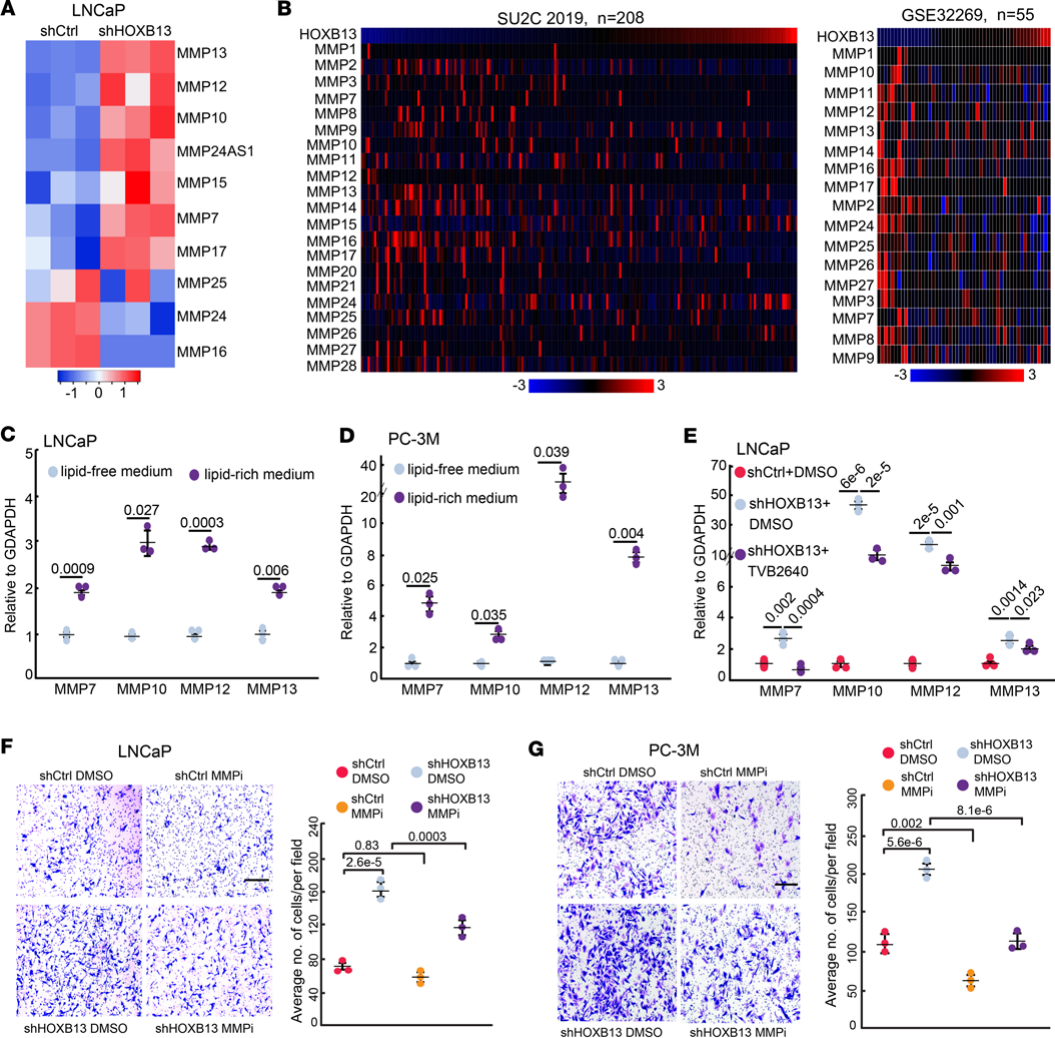
Lipid-induced MMP genes mediate HOXB13-loss-induced cell motility. (**A**) Heatmap showing MMP expression in LNCaP RNA-seq with control or *HOXB13* KD. All expressed MMPs in LNCaP cells were included in analysis. Color bar: *z*-score. (**B**) Heatmaps showing expression of MMPs in the indicated PCa patient data sets with samples ordered by HOXB13 level (top row). All the expressed MMPs in patients were included in analysis. Color bar: *z*-score. (**C** and **D**) RT-qPCR analysis of *MMP* expression in LNCaP (**C**) and PC-3M (**D**) cells cultured in lipid-free medium or lipid-rich medium (2% lipid mixture). Data were normalized to *GAPDH* (mean ± SEM, *n* = 3). *P* values were calculated by unpaired, 2-sided *t* test. (**E**) RT-qPCR analysis of *MMP* expression in LNCaP cells with sh*HOXB13* and/or TVB-2640 treatment. Data were normalized to *GAPDH* (mean ± SEM, *n* = 3). *P* values were calculated by unpaired, 2-sided *t* test. (**F** and **G**) Cell invasion assays of control or *HOXB13*-KD LNCaP (**F**) and PC-3M (**G**) cells treated with DMSO or MMPi. Representative images are shown (left), and the number of invaded cells is quantified (right). Scale bars: 50 μm. Quantification data are the mean ± SD of technical replicates from 1 of 2 (*n* = 2) independent experiments. *P* values were calculated by unpaired, 2-sided *t* test.

To connect HOXB13-loss-induced lipogenesis with PCa metastasis, we analyzed the expression of epithelial-mesenchymal transition (EMT) signature genes, which are well known to promote metastasis, in publicly available PCa datasets. We found that these genes were expressed at much higher levels in HOXB13-low compared with HOXB13-high tumors (Supplemental Figure 2A), indicating that HOXB13 loss may induce EMT gene expression. Further analysis of genes enriched in the EMT hallmark in our *HOXB13*-KD cells revealed increased expression of a few key EMT regulators, including *SNAI1/2* and *ZEB1*, and variable expression of others (Supplemental Figure 2, B and C). However, a significant upregulation of EMT effectors such as MMP genes, specifically *MMP7*, *MMP10*, *MMP12*, and *MMP13*, was observed (Figure 4A). Of these, MMP24 and MMP16 were expressed at very low levels in LNCaP to begin with and thus their reduction is negligible. These findings suggest that *HOXB13* KD may enhance cell motility primarily through MMP-mediated disruption of the extracellular matrix (ECM), rather than by broadly increasing PCa cell plasticity through EMT. Of clinical relevance, we validated elevated MMP expression in HOXB13-low compared with HOXB13-high PCa patient samples (Figure 4B). It has been shown that the expression of MMPs is regulated by lipids in adipose tissue. In line with this, we found that *MMP* gene expression was significantly increased upon stimulation by lipid mixture in LNCaP and PC-3M PCa cell lines (Figure 4, C and D). Critically, RT-qPCR confirmed their upregulation in PCa cells following *HOXB*13 KD, which was abolished by FASN inhibition using TVB-2640 (Figure 4E), suggesting that lipids, or fatty acids, increased in *HOXB13*-KD cells may induce *MMP* expression.

Next, we sought to determine whether the induction of MMPs is critical for the increased motility of *HOXB13*-depleted cells that we have previously reported (9). As multiple MMPs were upregulated in *HOXB13*-KD cells, we chose PF-00356231 hydrochloride, a broad-spectrum MMP inhibitor (MMPi), with potent activity against MMP12, MMP13, MMP9, MMP8, and MMP3 (32), to block these MMPs simultaneously. In vitro cell invasion assays revealed that *HOXB*13-KD-induced cell motility was significantly abolished by MMPi in both LNCaP and PC-3M cells (Figure 4, F and G), while cell proliferation was minimally affected (Supplemental Figure 2D). As a control, lipid-rich medium induced LNCaP cell invasion, which was profoundly attenuated by MMPi treatment (Supplemental Figure 2E). In summary, our data suggest that HOXB13-loss-induced lipogenesis promotes PCa cell invasion, at least in part, via MMPs.

### HOXB13-KD-induced lipid accumulation and cell invasion were abolished by p300/CBP inhibitors.

**Figure 5:**
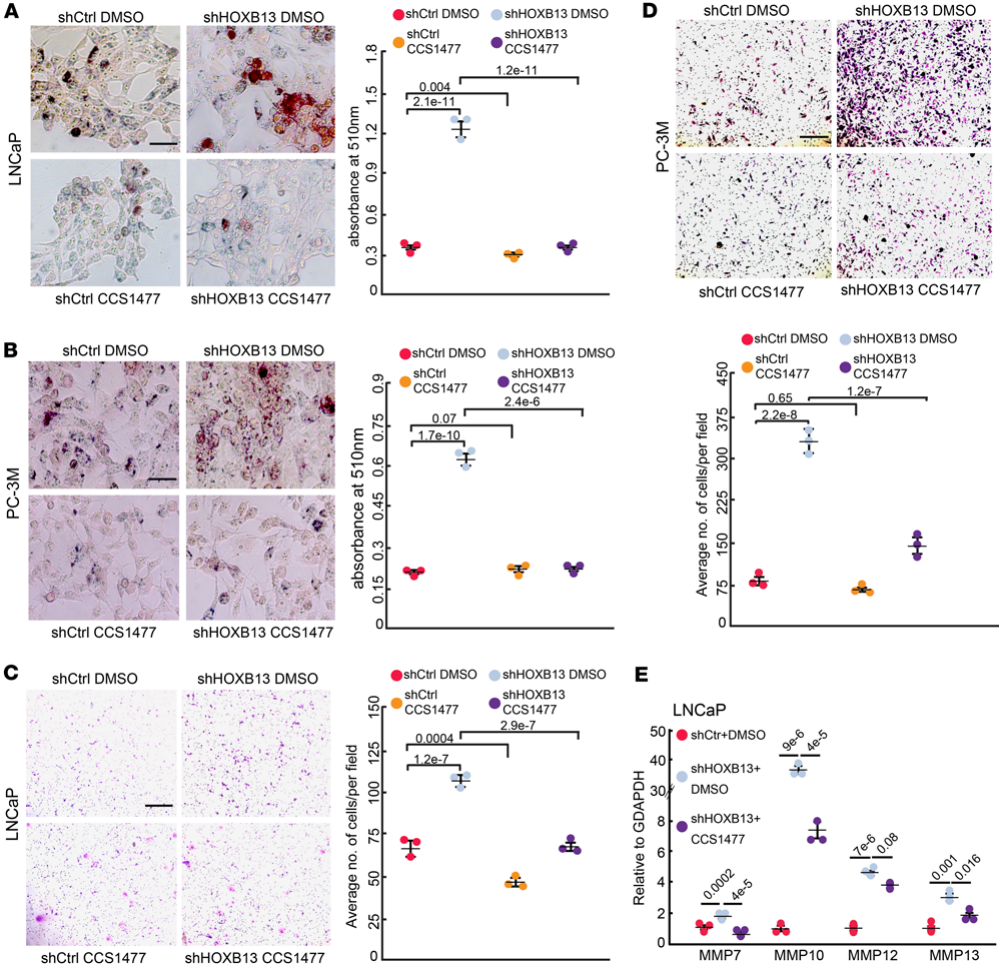
p300 and CBP inhibitors mitigate *HOXB13*-KD-induced lipid accumulation and cell invasion. (**A** and **B**) Representative images of ORO staining (left) and quantification (right) of neutral lipids in LNCaP (**A**) and PC-3M (**B**) cells with sh*HOXB13* and/or CCS1477 treatment. Scale bars: 30 μm. Quantification data are the mean ± SD of technical replicates from 1 of 2 (*n* = 2) independent experiments. *P* values were calculated by unpaired, 2-sided *t* test. (**C** and **D**) Cell invasion assays of control or *HOXB13*-KD LNCaP (**C**) and PC-3M (**D**) cells treated with DMSO or CCS1477. CCS1477: 250 nM for LNCaP, 500 nM for PC-3M. Representative images are shown (left/top), and the number of invaded cells is quantified (right/bottom). Scale bar: 50 μm. Quantification data are the mean ± SD of technical replicates from 1 of 2 (*n* = 2) independent experiments. *P* values were calculated by unpaired, 2-sided *t* test. (**E**) RT-qPCR analysis of *MMP* expression in LNCaP cells with sh*HOXB13* and/or CCS1477 treatment. Data were normalized to *GAPDH* (mean ± SEM, *n* = 3). *P* values were calculated by unpaired, 2-sided *t* test.

To discover therapeutic approaches to target HOXB13 loss in metastatic PCa, we tested the clinically available p300/CBP inhibitor CCS1477, since p300 and CBP are required for HOXB13-loss-induced lipogenic gene expression. We first performed Oil Red O (ORO) staining to detect neutral lipids in control and *HOXB13*-KD LNCaP cells treated with CCS1477. Consistent with our previous report (9), the depletion of *HOXB13* induced massive lipid accumulation in LNCaP cells, which, importantly, was drastically reduced upon treatment with CCS1477 (Figure 5A). Similar results were observed in the CRPC cell line 22Rv1 and the AR-negative PC-3M cells (Figure 5B and Supplemental Figure 3, A and B), suggesting an AR-independent mechanism of action. Altogether, our data demonstrated that p300/CBP inhibition significantly abolished HOXB13-loss-induced lipid accumulation in PCa cells, consistent with the transcriptome data shown earlier.

Accordingly, in vitro invasion assays revealed that CCS1477 significantly reduced *HOXB13*-KD-induced cell invasion in both LNCaP and PC-3M cells (Figure 5, C and D). Importantly, this effect was partially rescued by lipid supplementation (Supplemental Figure 3C), further supporting the idea that CCS1477 suppresses *HOXB13*-KD cell invasion by blocking lipid biosynthesis. Notably, although CCS1477 also decreased control cell invasion, the magnitude of changes was much less than that of the *HOXB13*-KD cells, suggesting an increased sensitivity of HOXB13-low cells to CCS1477. Moreover, *HOXB13*-KD cells did not exhibit increased sensitivity to CCS1477 in terms of cell proliferation, suggesting that the effect of p300/CBP inhibition in this context is specific to cell motility rather than growth (Supplemental Figure 3D). As our earlier data indicated that HOXB13-loss-induced cell motility is dependent on MMP genes, we examined whether MMP genes are involved in the cellular response to CCS1477. RT-qPCR analysis showed that the upregulation of *MMPs* in *HOXB13*-KD cells was drastically reduced by CCS1477 treatment (Figure 5E). In summary, our data support that *HOXB13*-KD-induced lipid accumulation and cell invasion can be effectively targeted by p300/CBP inhibitors, likely by suppressing MMP genes.

### Pharmacological inhibitors of p300/CBP abolished HOXB13-loss-induced tumor metastasis.

**Figure 6:**
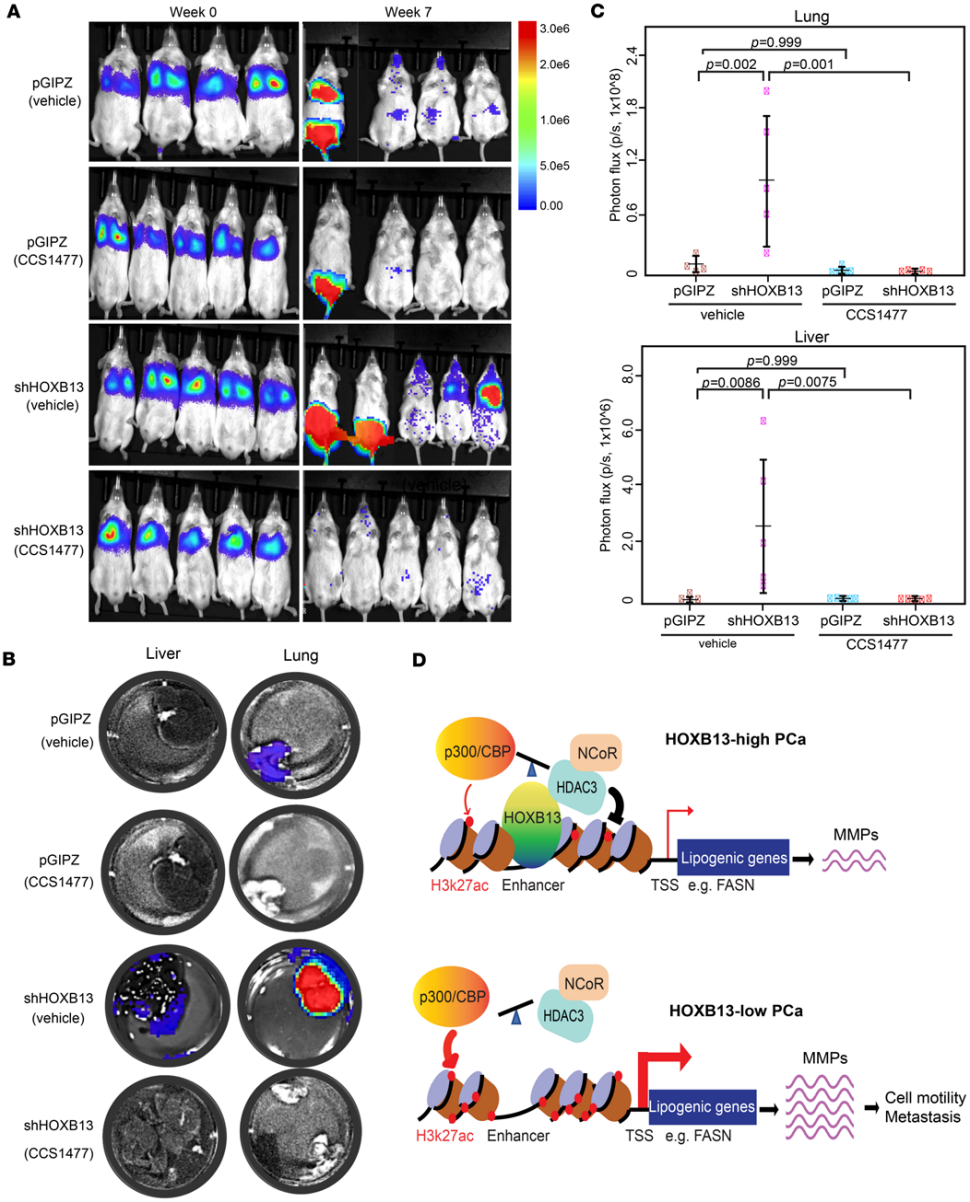
Pharmacological inhibitors of p300 and CBP abolished HOXB13-low tumor metastasis. (**A**) IVIS imaging of intravenous PC-3M xenograft tumors at weeks 0 (left) and 7 (right) after inoculation. Mice (*n* = 4 mice for pGIPZ vehicle group, *n* = 5 mice for the remaining groups) started treatment with CCS1477 on day 10. Heatmap shows IVIS signal intensity color scale. (**B** and **C**) Representative ex vivo IVIS images (**B**, *n* = 4 mice for pGIPZ vehicle group, *n* = 5 mice for the remaining groups) and quantifications (**C**) of PC-3M tumor metastasis to the lung and liver. Heatmap shows IVIS signal intensity color scale. Data are mean ± SD. Indicated *P* values were calculated by unpaired, 2-sided *t* test. (**D**) Graphical model illustrating HOXB13 as a molecular coordinator balancing HDAC3/NCoR and p300/CBP to fine-tune lipogenic gene expression in PCa. In HOXB13-high PCa cells, HOXB13 recruits the HDAC3-NCoR complex to lipogenic enhancers, catalyzing H3K27ac deacetylation and maintaining lipogenic gene expression at a basal level. In HOXB13-low PCa, this balance is disrupted — reduced recruitment of HDAC3 and NCoR leads to increased p300 and CBP binding and elevated H3K27ac levels at lipogenic enhancers. This results in aberrant activation of lipogenic programs, and induction of EMT-promoting genes such as MMPs.

Next, we evaluated whether p300/CBP inhibition can attenuate HOXB13-loss-induced tumor metastasis in vivo. As our earlier results showed that *HOXB13* KD had minimal impact on PC-3M cell proliferation, we generated intravenous PCa xenograft models by inoculating luciferase-labeled PC-3M cells with either control (pGIPZ) or *HOXB13* KD into nude SCID mice through tail vein injection and monitored tumor metastasis. In vivo imaging system (IVIS) confirmed comparable tumor cell uptake in the lungs between groups immediately after tail vein injection (Figure 6A, week 0). At 10 days after PCa cell inoculation, mice bearing control or *HOXB13*-KD tumors were randomized to receive either vehicle (5% DMSO, 95% methylcellulose) or CCS1477 (20 mg/kg, daily) for 7 weeks by oral gavage. At week 7 after inoculation, IVIS showed apparent metastasis in 4 out of 5 *HOXB13*-KD mice — 2 in the lung and the other 2 in the prostate. In contrast, only 1 mouse in the control group developed detectable metastatic tumors (in the lung and prostate), confirming that *HOXB13* KD increased tumor metastasis (Figure 6A, week 7). Importantly, none of the CCS1477-treated *HOXB13*-KD mice developed tumor metastasis, strongly supporting the idea that CCS1477 treatment eliminated *HOXB13*-KD-induced metastasis. Furthermore, the body weight of mice was not significantly affected by the drug (Supplemental Figure 4A), indicating the tolerability of the drug. Distant organs were dissected from the mice at the endpoint. IVIS imaging revealed that more tumor cells metastasized to distant organs, such as the liver, lung, rib, hind leg, and prostate, in the HOXB13-KD group compared with the control and CCS1477-treated mice (Figure 6, B and C, and Supplemental Figure 4, B–D). In summary, these data demonstrate that CCS1477 can effectively suppress HOXB13-loss-induced PCa metastasis in vivo without apparent toxicity, and thus may represent a promising therapeutic strategy for metastatic PCa with low HOXB13 expression.

## Discussion

Our previous study revealed that HOXB13 interacts with the HDAC3-NCoR complex and recruits them to lipogenic enhancers to catalyze histone deacetylation, resulting in the suppression of lipogenic programs (9). Accordingly, HOXB13 loss results in an enhanced lipogenic program in PCa, which has been associated with tumor metastasis. However, it remained unclear whether active catalysis of histone acetylation and the enzymes p300 and CBP are directly involved in this process. Previous studies have shown that the HDAC3-NCoR co-repressor complex and p300/CBP function antagonistically to regulate steady-state histone acetylation dynamics (10, 13). These complexes are recruited and co-localized with lineage-specific or oncogenic transcription factors at enhancers, where they fine-tune lineage-determined gene expression or drive oncogenic transcriptional programs (33–35), respectively. In agreement with such dogma, here, we found that p300 and CBP co-occupy HOXB13/HDAC3 shared binding sites and that the loss of HOXB13 disrupts the balance between HDAC3 and p300/CBP, leading to reduced HDAC3 recruitment, increased p300 and CBP occupancy, elevated H3K27ac levels, and aberrant activation of lipogenic programs (Figure 6D). These findings position HOXB13 as a molecular coordinator that modulates HDAC3 and p300 and CBP activity to fine-tune lipid-related transcription in PCa. Furthermore, the increased p300 and CBP binding at lipogenic enhancers in *HOXB13*-depleted cells may involve other lipid-regulating transcription factors, such as SREBPs that are also increased by *HOXB13* KD (9). SREBPs are known to interact with p300 and CBP and recruit them to lipogenic gene loci, such as *FASN*, to induce their expression in liver and adipose tissue in response to insulin (36). Whether SREBPs similarly contribute to lipogenic enhancer activation in *HOXB13*-deficient PCa cells remains an important question for future investigation.

Clinically, lipid dysregulation and accumulation have been strongly associated with PCa aggressiveness. HFD promotes PCa metastasis in preclinical models (26), and aberrant accumulation of lipid droplets has been seen in high-grade, metastatic PCa and circulating prostate tumor cells (18). However, the molecular mechanisms linking lipid metabolism to metastasis remain elusive. In the current study, we identified MMP genes, key effectors of EMT and cell motility, as downstream targets induced in *HOXB13*-depleted cells (Figure 6D). This induction might be caused by lipid accumulation in the cells but might also be due to the regulation by upstream lipogenic enzymes. Importantly, both p300/CBP inhibition and FASN inhibition suppressed MMP expression and abolished HOXB13-loss-induced cell motility, suggesting a lipid/MMP/cell invasion axis (Figure 6D). Supporting this, a recent study showed that fatty acids, such as palmitate, promote murine lung cancer cell metastasis via acetylation of NF-κB subunit p65, resulting in activation of NF-κB signaling and upregulation of prometastatic genes, such as *Mmp9* and *Tnfaip2* (37). Whether a similar mechanism underlies lipid-induced MMP activation in our models warrants further investigation.

Several p300 and CBP inhibitors have been developed, including CCS1477. Notably, CCS1477 has shown potent activity in suppressing PCa cell growth in vitro and in vivo, in part by downregulating AR and MYC expression, and is currently under evaluation in a first-in-human phase I clinical trial for advanced PCa (16). In the current study, we found that *HOXB13*-KD-induced lipogenic programs are dependent on p300 and CBP activity. Notably, approximately 60% of CCS1477-repressed genes in HOXB13-KD cells were induced by HOXB13 KD, and these genes were enriched in lipogenesis, suggesting that they are the primary targets of CCS1477 in this context. Accordingly, treatment with CCS1477 effectively abolished *HOXB13*-KD-induced lipid accumulation, and thus PCa cell invasion in vitro and prostate tumor metastasis in vivo. While our in vitro studies analyzed both AR-positive and AR-negative cell lines, supporting the generality of our findings, our in vivo investigation of CCS1477 was limited to AR-negative PC-3M cells. This limitation can be addressed by broader testing in additional models in the future. Clinically, we observed that HOXB13 expression is significantly reduced in LN metastases compared with paired primary tumors from the same patient before any hormonal treatment, suggesting that HOXB13 loss is associated with metastatic progression rather than disrupted androgen signaling. While our current cohort included only lymph node metastases, previous studies of metastatic CRPC (including liver and bone metastases) similarly demonstrated consistent HOXB13 downregulation (9), supporting its broader role in advanced metastasis beyond LNs. Future analyses of HOXB13 in metastatic tumors, such as liver, lung, and bone, of HN PCa relative to primary sites will further clarify this point. In summary, our findings suggest that CCS1477 or other p300/CBP-targeting agents may be promising therapeutic options for patients with HOXB13-low, metastatic, hormone-sensitive PCa.

## Methods

### Sex as a biological variable.

In this study, only male mice (NOD SCID mice, 6–7 weeks old) were used. PCa is a male-specific disease, and thus, male mice were selected to accurately model the biological and clinical characteristics of the disease. The use of male mice ensures relevance to human PCa, and findings are not expected to vary significantly across sexes, as PCa primarily affects males. This choice aligns with the biological specificity of the disease.

### Cell lines, antibodies and chemical reagents.

PCa cell lines LNCaP, 22Rv1, PC-3M and human embryonic kidney cell line HEK293T were obtained from the American Type Culture Collection (ATCC) and cultured in either RPMI 1640 or DMEM with 10% FBS and 1% penicillin/streptomycin. Cell lines were either newly acquired from ATCC or authenticated within 6 months of growth and cells under culture are frequently tested for potential mycoplasma contamination. The antibodies used in this study include HOXB13 (Cell Signaling Technology, 90944T), PSA (Cell Signaling Technology, 2475T), p300 (Cell Signaling Technology, 54062S), CBP (Cell Signaling Technology, 7389S), FASN (Santa Cruz Biotechnology, sc-48357) for WB application, and p300 (Bethyl, A300-358A) and H3K27ac (Cell Signaling Technology, 8173S) for ChIP application. The small-molecule inhibitors used were CCS1477 (Chemietek, CT-CCS1477) and PF-00356231 hydrochloride (MedChemExpress, HY-114091).

### Constructs and lentivirus.

The shRNAs targeting *CBP* and *p300* were cloned into pLKO.1-TRC lentiviral vector (Addgene, 10878). The pGIPZ lentiviral vector expressing an shRNA targeting *HOXB13* (clone ID: V3LHS_403019), along with the corresponding control vector, was obtained from Open Biosystems. All plasmid constructs were verified by Sanger sequencing, and the oligonucleotide sequences used in this study are listed in Supplemental Table 2. Lentiviruses were generated by co-transfecting HEK293T cells with psPAX2, pMD2.G, and the respective shRNA plasmid at a ratio of 2:1:1 using a standard PEI transfection protocol. Viral supernatants were collected 48 hours after transfection, filtered through a 0.45 μm filter, and used to infect PCa cells in the presence of 8 μg/mL polybrene to enhance transduction efficiency.

### RNA extraction, RT-qPCR, and RNA-seq.

Total RNA was extracted using the Nucleospin RNA kit (Takara) according to the manufacturer’s recommended protocol. For cDNA synthesis, 500 ng of RNA was reverse transcribed using the ReverTra Ace qPCR RT Master Mix Kit (Diagnocine), also according to the manufacturer’s protocol. Quantitative PCR (qPCR) was carried out using 2× Universal SYBR Green Fast qPCR Mix (Abclonal, RK21203) on a QuantStudio 3 Real-Time PCR System (Thermo Fisher Scientific). Primer sequences used for qPCR are listed in Supplemental Table 2. For RNA-seq, total RNA was isolated as described above, with experiments performed with biological duplicates or triplicates. High-quality, DNA-free RNA (0.5 μg) was used to prepare RNA-seq libraries using the NEBNext Ultra RNA Library Prep Kit (New England Biolabs), in accordance with the manufacturer’s protocol. Libraries were assessed for quality, ensuring a fragment size distribution between 250 and 400 bp, absence of adapter contamination, and no signs of RNA degradation. Qualified libraries were quantified using the Library Quantification Kit for Illumina (Kapa Biosystems, KK4603), pooled to a final concentration of 10 nM, and sequenced using paired-end on the Illumina NovaSeq 6000 platform.

### ChIP and ChIP-seq.

ChIP and ChIP-seq were performed using a previously described protocol (9, 38), with the following modifications. For H3K27ac ChIP, PCa cells were crosslinked in 1% formaldehyde (Thermo Fisher Scientific, 28908) for 10 minutes at room temperature with gentle rotation, followed by quenching with 0.125 M glycine for 5 minutes. A total of 2 million cells were used per ChIP reaction. For p300 ChIP, double crosslinking was employed. Ten million cells were first treated with 2 mM disuccinimidyl glutarate (DSG, Pierce) for 10 minutes at room temperature, followed by crosslinking with 1% formaldehyde for an additional 10 minutes. The crosslinking reaction was quenched with 0.125 M glycine for 5 minutes at room temperature. For ChIP-seq, *Drosophila* S2 cells were used as a spike-in control to normalize ChIP signals, following the protocol provided by Active Motif. Primer sequences used for ChIP-qPCR are listed in Supplemental Table 2.

### RNA-seq and ChIP-seq analysis.

For RNA-seq, raw sequencing reads were aligned to the NCBI human reference genome GRCh38 using STAR (https://github.com/alexdobin/STAR). Gene-level raw counts were calculated, and FPKM values (fragments per kilobase of transcript per million mapped reads) were computed using an in-house Perl script (Supplemental Methods). Differentially expressed genes (DEGs) were identified using DESeq2 (https://bioconductor.org/packages/release/bioc/html/DESeq2.html) with the default Wald’s test in a pairwise comparison.

For ChIP-seq analysis, sequencing reads were aligned to the human reference genome hg38 using Bowtie2 (v2.0.5; https://bowtie-bio.sourceforge.net/bowtie2/index.shtml). FastQC (v0.12; https://www.bioinformatics.babraham.ac.uk/projects/fastqc/) was used to assess sequencing quality. Adapter sequences were removed using Trim Galore (v0.6.5; https://www.bioinformatics.babraham.ac.uk/projects/trim_galore/). Non–uniquely mapped reads, duplicated reads, and genomic blacklisted regions were excluded. The remaining high-quality reads were used to identify ChIP-enriched binding peaks using HOMER (v4.11; http://homer.ucsd.edu/homer/) with default parameters. Heatmaps and signal density plots of ChIP-seq signals were generated using deepTools (https://deeptools.readthedocs.io/en/latest/). For H3K27ac ChIP-seq, *Drosophila* S2 spike-in normalization was applied to account for technical variability.

### Cell invasion, colony formation assay, and ORO staining of neutral lipids.

Cell invasion assays were performed as previously described (9), with the following modifications. LNCaP (30,000 cells) or PC-3M (15,000 cells) were seeded into the upper chambers of Transwell inserts. The upper chamber contained complete growth medium with 1% FBS, while the lower chamber was filled with medium containing 20% FBS to serve as a chemoattractant. After 48 hours of incubation, invading cells were fixed, stained, and quantified. The colony formation assays and ORO staining assays of LNCaP and PC3M cells were performed as previously reported (9).

### Murine intravenous xenograft studies.

All mice were housed in a specific pathogen–free (SPF) facility under controlled conditions (20°C–23°C, 40%–60% humidity, 12-hour light/12-hour dark cycle). NOD SCID male mice (6–7 weeks old) were obtained from Charles River Laboratories. Intravenous injection was performed as previously described (9). Briefly, luciferase-labeled PC-3M cells stably expressing either control shRNA or HOXB13-targeting shRNA (2 × 10^6^ cells in 200 μL of 1× PBS) were injected into the tail vein. Ten days after injection, mice were randomly assigned to receive either vehicle (5% DMSO, 95% methylcellulose) or CCS1477 (20 mg/kg) via daily oral gavage for 7 weeks. Tumor metastasis was monitored weekly using the Lago Bioluminescence/Fluorescence Imaging System (Spectral Instruments Imaging) at the Northwestern University Core Facility, following the manufacturer’s protocol. For ex vivo IVIS imaging, mice were euthanized 10 minutes after intraperitoneal injection of d-luciferin, and major organs, including liver, lung, rib, and hind leg, were immediately harvested and imaged.

### IHC and TMA analysis.

Two PCa tissue microarrays (TMAs), constructed with approval from their respective Institutional Review Board, were utilized in this study. The Emory cohort consisted of 56 high-grade primary PCa and 56 LN-metastatic PCa samples, all derived from patients with no prior hormone therapy. Among these, 42 cases included matched primary and LN-metastatic tumor tissues from the same individuals. The NYU cohort comprised 30 HN primary PCa samples, 5 samples from patients who received hormone therapy for less than 2 months (HR<2M), and 45 samples from patients treated for more than 2 months (HR>2M). TMAs were constructed by the Prostate Cancer Biorepository Network (PCBN) at New York University, with approval from the NYU Institutional Review Board. HOXB13 IHC was performed using a validated antibody (Cell Signaling Technology, 90944S) by the Emory University Pathology Core and the Northwestern University Pathology Core. Whole-slide images were captured using the Zeiss Axioscan 7 at the Emory University Imaging Core and analyzed with ZEN lite software (Zeiss). HOXB13 staining intensity was blindly scored by a board-certified pathologist on a scale of 0 to 3, corresponding to negative, weak, moderate, or strong staining, and multiplied by the percentage of positive tumor cells to generate a final IHC score.

### Statistics.

For each independent in vitro experiment, at least 3 technical replicates were used. Most in vitro experiments were repeated independently three times, and some were repeated twice. Two-tailed, unpaired Student’s *t* tests were used to assess statistical significances in RT-qPCR experiments and cell-based functional assays. One-way analysis of variance (ANOVA) was used to determine statistically significant differences across treatment groups.

### Study approval.

Our research complied with all relevant ethical regulations. Mouse handling and experimental procedures were approved by the Institutional Animal Care and Use Committee at Northwestern University in accordance with the US NIH *Guide for the Care and Use of Laboratory Animals* (National Academies Press, 2011) and the Animal Welfare Act.

### Data availability.

All RNA-seq and ChIP-seq data generated in this study are available in the NCBI Gene Expression Omnibus (GEO GSE296250). Values for all data points in graphs are reported in the Supporting Data Values file.

## Author contributions

JY and XL conceived the project and designed the experiments. QC, XL, JCZ, and JY conducted bioinformatic and statistical analyses. XL, LP, ML, and SY performed experiments. XL, JM, and LRH performed HOXB13 IHC staining and scoring. MH and MAB provided guidance on the clinical implications of the study. XL and JY wrote the manuscript.

## Funding support

This work is the result of NIH funding, in whole or in part, and is subject to the NIH Public Access Policy. Through acceptance of this federal funding, the NIH has been given a right to make the work publicly available in PubMed Central.

- NIH/National Cancer Institute grant R01CA257446 (to JY).
- Department of Defense grant PC210266 (to JY).
- Northwestern University Pathology Core Facility, Emory University Pathology Core Facility, and Emory Imaging Core Facility (partial support).

## Supplementary Material

Supplemental data

Unedited blot and gel images

Supporting data values

## Version 1 11/24/2025

Electronic publication
