# Supplementary material for: Targeting p300 and CBP abolishes HOXB13-loss-induced lipogenesis and tumor metastasis
Source: JCI Insight. 2025 Nov 24;10(22):e195743. doi: 10.1172/jci.insight.195743 (PMC12643514; doi:10.1172/jci.insight.195743)
Supplement: Supplemental data [file jciinsight-10-195743-s111.pdf]

Fig.S 1

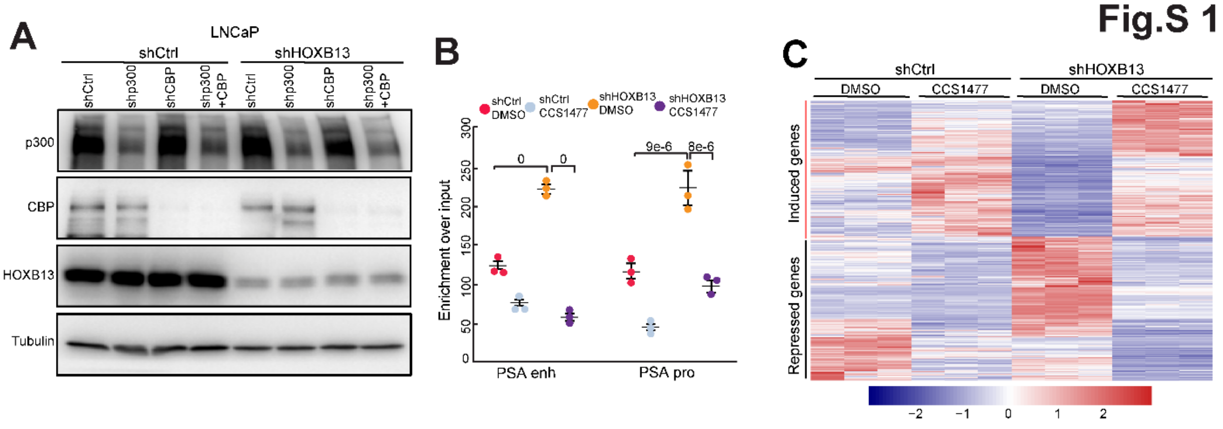

Figure S1. p300/CBP are required for HOXB13 loss-induced lipogenic program

A. WB analysis of p300, CBP, and HOXB13 knockdown (KD) efficiency in LNCaP cells with indicated treatment.

B. H3K27ac ChIP-QPCR of PSA enhancer (enh) and promoter (pro) in LNCaP cells with *HOXB13* KD and/or CCS1477 treatment. Data were normalized to 2% of input DNA. Shown are mean ±sem of technical replicates from one representative experiment of three. Statistical significance was determined by one-way ANOVA followed by Tukey's multiple comparisons test.

C. Heatmap showing CCS1477-induced and -repressed genes in control or *HOXB13*-KD LNCaP cells. CCS1477-regulated genes were identified by DESeq2 with FC⩾1.5, adjusted p<0.05. Color bar: z-score.

Fig.S2

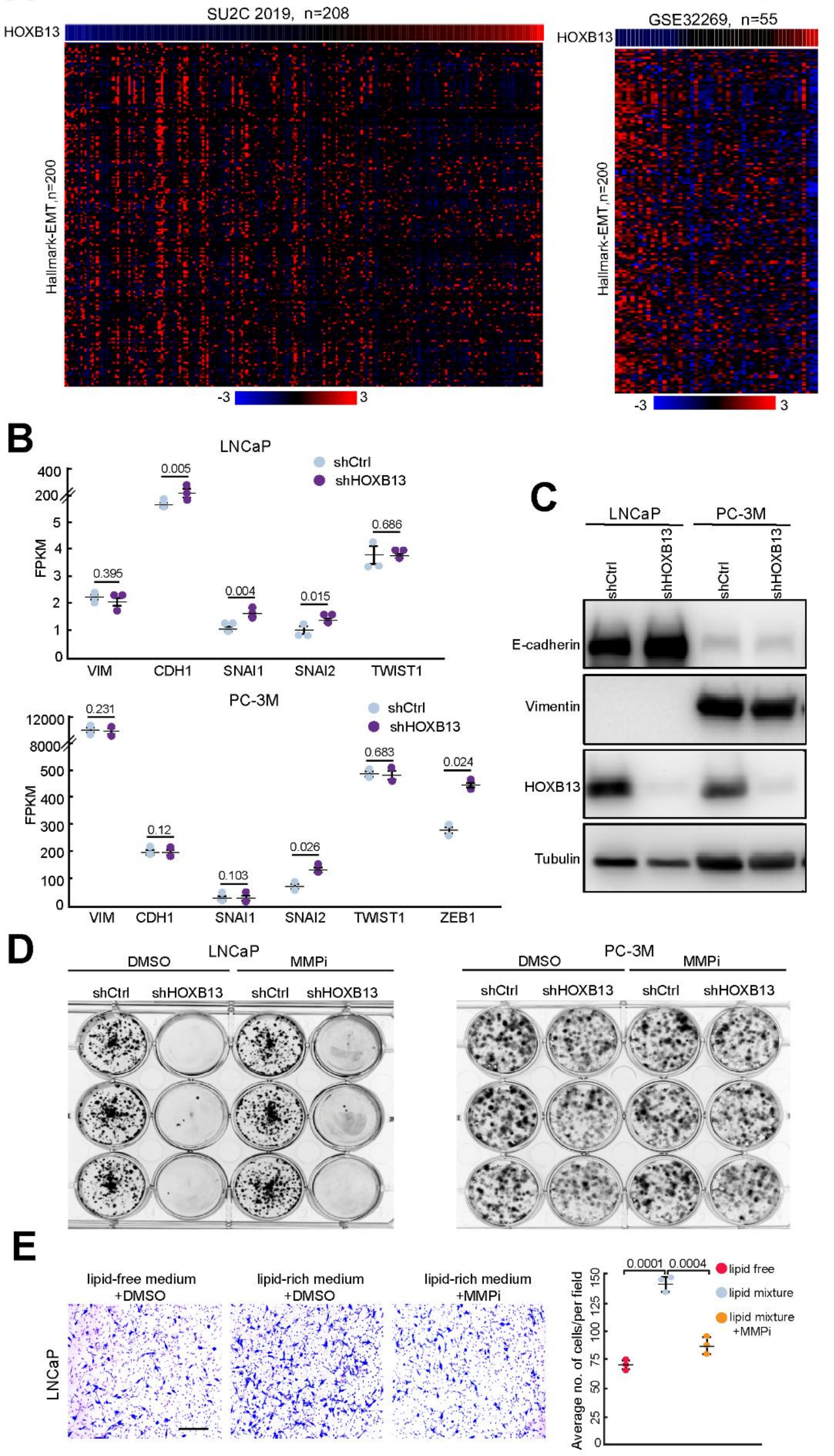
A
SU2C 2019, n=208
HOXB13
Hallmark-EMT,n=200
-3
3
GSE32269, n=55
HOXB13
Hallmark-EMT,n=200
-3
3
B
LNCaP
shCtrl
shHOXB13
FPKM
0.005
0.395
0.004
0.015
0.686
VIM
CDH1
SNAI1
SNAI2
TWIST1
PC-3M
shCtrl
shHOXB13
0.231
0.12
0.103
0.026
0.683
0.024
VIM
CDH1
SNAI1
SNAI2
TWIST1
ZEB1
C
LNCaP
PC-3M
shCtrl
shHOXB13
shCtrl
shHOXB13
E-cadherin
Vimentin
HOXB13
Tubulin
D
LNCaP
DMSO
MMPi
shCtrl
shHOXB13
shCtrl
shHOXB13
PC-3M
DMSO
MMPi
shCtrl
shHOXB13
shCtrl
shHOXB13
E
lipid-free medium +DMSO
lipid-rich medium +DMSO
lipid-rich medium +MMPi
LNCaP
Average no. of cells/per field
0.0001
0.0004
lipid free
lipid mixture
lipid mixture +MMPi

Figure S2. Matrix metalloproteinases (MMPs) are induced upon HOXB13 loss to increase cell motility

A. Heatmap showing HALLMARK_EPITHELIAL_MESENCHYMAL_TRANSITION (Hallmark-EMT) signature genes in the indicated PCa patient data sets with samples ordered by HOXB13 level (top row). The Hallmark-EMT signatures were downloaded from MSigDB. Color bar: z-score.

B. RNA-seq analysis of EMT regulators and markers (CDH1 and VIM) in LNCaP (top) and PC-3M (bottom) cells with control or *HOXB13* KD.

C. WB analysis of EMT markers (CDH1 and VIM) in LNCaP and PC-3M cells with control or *HOXB13* KD.

D. Colony formation assays of LNCaP (left) and PC-3M (right) cells with sh*HOXB13* and/or MMPi treatment.

E. Cell invasion assays of LNCaP cells cultured in lipid-free medium, lipid-rich medium (2% lipid mixture) or lipid-rich medium (2% lipid mixture) plus MMP inhibitor (MMPi). Representative images are shown (left), and the number of invaded cells is quantified (right). Scale bar, 50 μm. Statistical significance was determined by one-way ANOVA followed by Tukey's multiple comparisons test.

Fig.S3

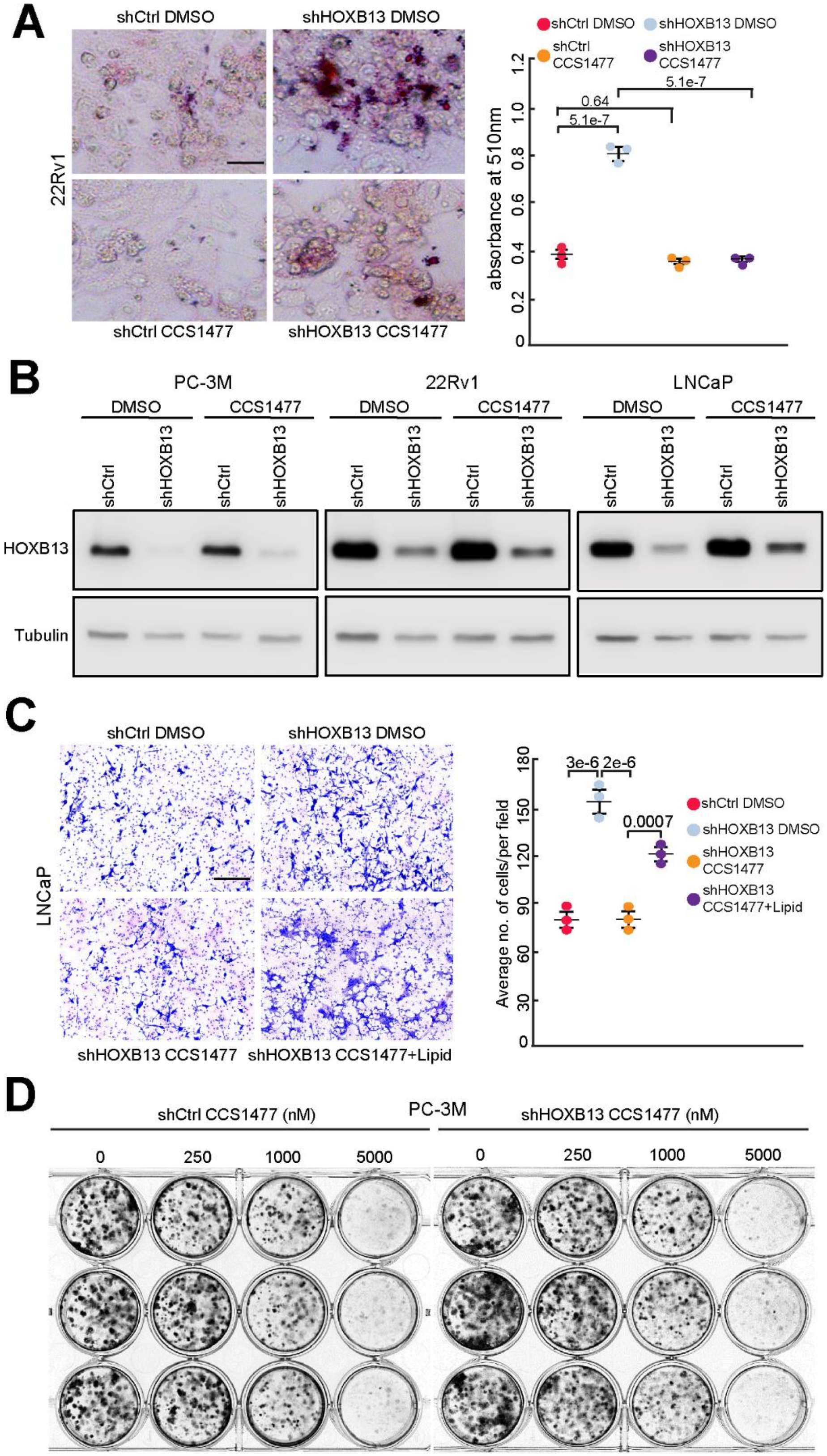
A
shCtrl DMSO
shHOXB13 DMSO
22Rv1
shCtrl CCS1477
shHOXB13 CCS1477
shCtrl DMSO
shHOXB13 DMSO
shCtrl CCS1477
shHOXB13 CCS1477
absorbance at 510nm
5.1e-7
0.64
5.1e-7
B
PC-3M
22Rv1
LNCaP
DMSO
CCS1477
shCtrl
shHOXB13
HOXB13
Tubulin
C
shCtrl DMSO
shHOXB13 DMSO
LNCaP
shHOXB13 CCS1477
shHOXB13 CCS1477+Lipid
Average no. of cells/per field
3e-6
2e-6
0.0007
shCtrl DMSO
shHOXB13 DMSO
shHOXB13 CCS1477
shHOXB13 CCS1477+Lipid
D
PC-3M
shCtrl CCS1477 (nM)
shHOXB13 CCS1477 (nM)
0
250
1000
5000

Figure S3. p300/CBP inhibitors mitigate *HOXB13*-KD-induced lipid accumulation and cell invasion

A. Representative images of ORO staining (left) and quantification (right) of neutral lipids in 22Rv1cells with sh*HOXB13* and/or CCS1477 treatment. Scale bar, 30 µm. Quantification data are the mean ± s.d. of technical replicates from one of two (n = 2) independent experiments. Statistical significance was determined by one-way ANOVA followed by Tukey's multiple comparisons test.

B. WB analysis of *HOXB13* KD efficiency in LNCaP, 22Rv1 and PC-3M cells.

C. Cell invasion assays of control or *HOXB13*-KD LNCaP cells treated with DMSO or CCS1477 or CCS1477 plus lipid mixture. Representative images are shown (left), and the number of invaded cells is quantified (right). Scale bar, 50 µm. Statistical significance was determined by one-way ANOVA followed by Tukey's multiple comparisons test.

D. Colony formation assays of control (shCtrl) or *HOXB13*-KD (sh*HOXB13*) PC-3M cells treated with indicated concentration of CCS1477.

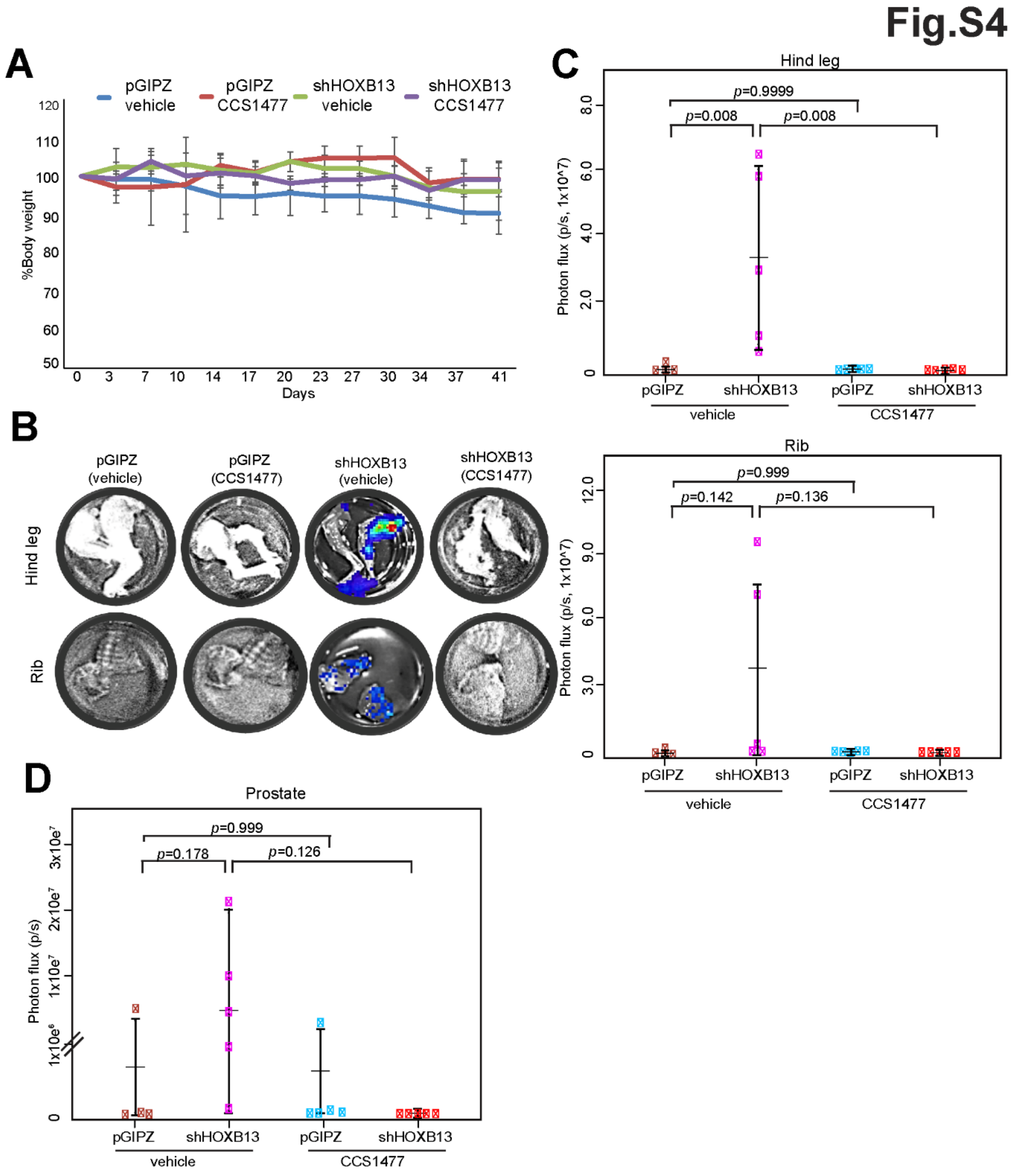

Figure S4. Therapeutic targeting of HOXB13-low tumors with p300/CBP inhibitor

A. Body weight analysis in mice inoculated by control or *HOXB13*-KD PC-3M cells and treated with vehicle or CCS1477. Data in each time point are mean ±s.d. Y-axis shows the percentage of body weight change.

B-D. Representative *ex vivo* IVIS images (**B**) and quantifications (**C-D**) of PC-3M tumor metastasis to the hind leg, rib and prostate. Heatmap shows IVIS signal intensity color scale.

Statistical significance was determined by one-way ANOVA followed by Tukey's multiple comparisons test.

Supplemental methods

FPKM (fragments per kilobase of transcript per million mapped reads) calculation with in-house Perl script

```
my @files = <*ReadsPerGene.out.tab>;

$inputname1 = 'igenome_gene_name_length.txt';
$inputname2 = 'sample_read_mapping.txt';

my %gene_hash;
my %mapping_hash;
my $mreads;

open(INPUTFILE1, $inputname1);
while($datline1 = <INPUTFILE1>)
{
	chomp($datline1);
	my @line1 = split("\t", $datline1);
#gid is actually gene_name, since it is unique, lazy to change code, gene_name used twice
	my $gid = $line1[0];
	my $genenamelength = "$line1[0]\t$line1[1]";
	$gene_hash{$gid} = $genenamelength;
}
close(INPUTFILE1);

open(INPUTFILE2, $inputname2);
while($datline2 = <INPUTFILE2>)
{
	chomp($datline2);
	my @line2 = split("\t", $datline2);
	my $filename = $line2[0];
	my $mappedreads = $line2[2];
	$mapping_hash{$filename} = $mappedreads;
}
close(INPUTFILE2);

foreach $file (@files)
{
	$fname = substr $file, 0, index($file, 'ReadsPerGene');
	if(exists($mapping_hash{$fname}))
	{
		$mreads = $mapping_hash{$fname};
```

```
	}

	open(OUTPUTFILE, '>', $fname.'_FPKM.txt');
	open(INPUTFILE, $file);

	while($datline = <INPUTFILE>)
	{
		next if 1..4;
		chomp($datline);
		my @line = split("\t", $datline);
		my $rgid = $line[0];
		my $rawcounts = $line[1];
		if(exists($gene_hash{$rgid}))
		{
			@gene = split("\t", $gene_hash{$rgid});
			$genename = $gene[0];
			$genelength = $gene[1];
			my $FPKM = sprintf "%.7f", ($rawcounts * 1000000000)/($mreads * 
$genelength);
			print OUTPUTFILE "$rgid\t$FPKM\n";
		}
		else
		{
			print OUTPUTFILE "$rgid\t\n";
		}
	}
	close(INPUTFILE);
	close(OUTPUTFILE);
}
```

Supplemental tables

Supplemental table 1. 45 genes involved in the fatty-acyl-CoA biosynthetic process and long-chain fatty-acyl-CoA biosynthetic process are enriched in region IV

Gene name	Gene/product name
ACAT1	Acetyl-CoA acetyltransferase, mitochondrial
ELOVL7	Very long chain fatty acid elongase 7
ACSL6	Long-chain-fatty-acid--CoA ligase 6
SLC27A2	Long-chain fatty acid transport protein 2
GCDH	Glutaryl-CoA dehydrogenase, mitochondrial
ELOVL1	Very long chain fatty acid elongase 1
ACSL4	Long-chain-fatty-acid--CoA ligase 4
ACSF3	Malonate--CoA ligase ACSF3, mitochondrial

CBR4	3-oxoacyl-[acyl-carrier-protein] reductase
ELOVL5	Very long chain fatty acid elongase 5
TECR	Very-long-chain enoyl-CoA reductase
ACACA	Acetyl-CoA carboxylase 1
HACD1	Very-long-chain (3R)-3-hydroxyacyl-CoA dehydratase 1
ELOVL2	Very long chain fatty acid elongase 2
ELOVL4	Very long chain fatty acid elongase 4
ACSL5	Long-chain-fatty-acid--CoA ligase 5
ACSL3	Fatty acid CoA ligase Acsl3
PPT1	Palmitoyl-protein thioesterase 1
HTD2	Hydroxyacyl-thioester dehydratase type 2, mitochondrial
ACSBG1	Long-chain-fatty-acid--CoA ligase ACSBG1
HACD2	Very-long-chain (3R)-3-hydroxyacyl-CoA dehydratase 2
PPT2	Lysosomal thioesterase PPT2
ACSBG2	Long-chain-fatty-acid--CoA ligase ACSBG2
FASN	Fatty acid synthase
ELOVL6	Very long chain fatty acid elongase 6
HSD17B12	Very-long-chain 3-oxoacyl-CoA reductase
ACSL1	Long-chain-fatty-acid--CoA ligase 1
ELOVL3	Very long chain fatty acid elongase 3
PDHA2	Pyruvate dehydrogenase E1 component subunit alpha, testis-specific form, mitochondrial
ACLY	ATP-citrate synthase
ACSS2	Acetyl-coenzyme A synthetase, cytoplasmic
ACSS1	Acetyl-coenzyme A synthetase 2-like, mitochondrial
MLYCD	Malonyl-CoA decarboxylase, mitochondrial
DLAT	Dihydrolipoyllysine-residue acetyltransferase component of pyruvate dehydrogenase complex, mitochondrial
PDHA1	Pyruvate dehydrogenase E1 component subunit alpha, somatic form, mitochondrial
ZNF516	Zinc finger protein 516
RORC	Nuclear receptor ROR-gamma
ABHD15	Protein ABHD15
SPTLC2	Serine palmitoyltransferase 2
PGRMC2	Membrane-associated progesterone receptor component 2
VPS13B	Intermembrane lipid transfer protein VPS13B
XBP1	X-box-binding protein 1
DYRK1B	Dual specificity tyrosine-phosphorylation-regulated kinase 1B
SOX8	Transcription factor SOX-8
SH3PXD2B	SH3 and PX domain-containing protein 2B

Supplemental table 2. Oligonucleotides that were used in this study

Primers:		
Name	Sequence (5′ to 3′)	Application
MMP7-F	GGAGGCATGAGTGAGCTACAG	RT-PCR
MMP7-R	GGCCAAAGAATTTTTGCATC	RT-PCR
MMP10-F	CACAGTTTGGCTCATGCCTA	RT-PCR
MMP10-R	AAGTTCATGAGCAGCAACGA	RT-PCR
MMP12-F	CTAGTGATCCAAAGGCCGTAAT	RT-PCR
MMP12-R	CACGGTAGTGACAGCATCAA	RT-PCR
MMP13-F	GGTTCCTGATGTGGGTGAAT	RT-PCR
MMP13-R	TGAATGCCTTTTCGACTTCA	RT-PCR
KLK3-AF	GCCTGGATCTGAGAGAGATATCATC	ChIP-PCR
KLK3-AR	ACACCTTTTTTTTTCTGGATTGTTG	ChIP-PCR
FASN-AF	CAGAAGAGTAAACGCAGGAGAA	ChIP-PCR
FASN-AR	CCTCACTTTAGGACCAGGAAAC	ChIP-PCR
shRNAs:		
Name	Sequence (5′ to 3′)	
shp300	AAGCTACTGAAGATAGATTAATA	
shCBP	GCAAGACATCCCGAGTCTATA	
