# Supplementary figures and images for: Targeting p300 and CBP abolishes HOXB13-loss-induced lipogenesis and tumor metastasis
Source: JCI Insight. 2025 Nov 24;10(22):e195743. doi: 10.1172/jci.insight.195743 (PMC12643514; doi:10.1172/jci.insight.195743)

Figure 2D

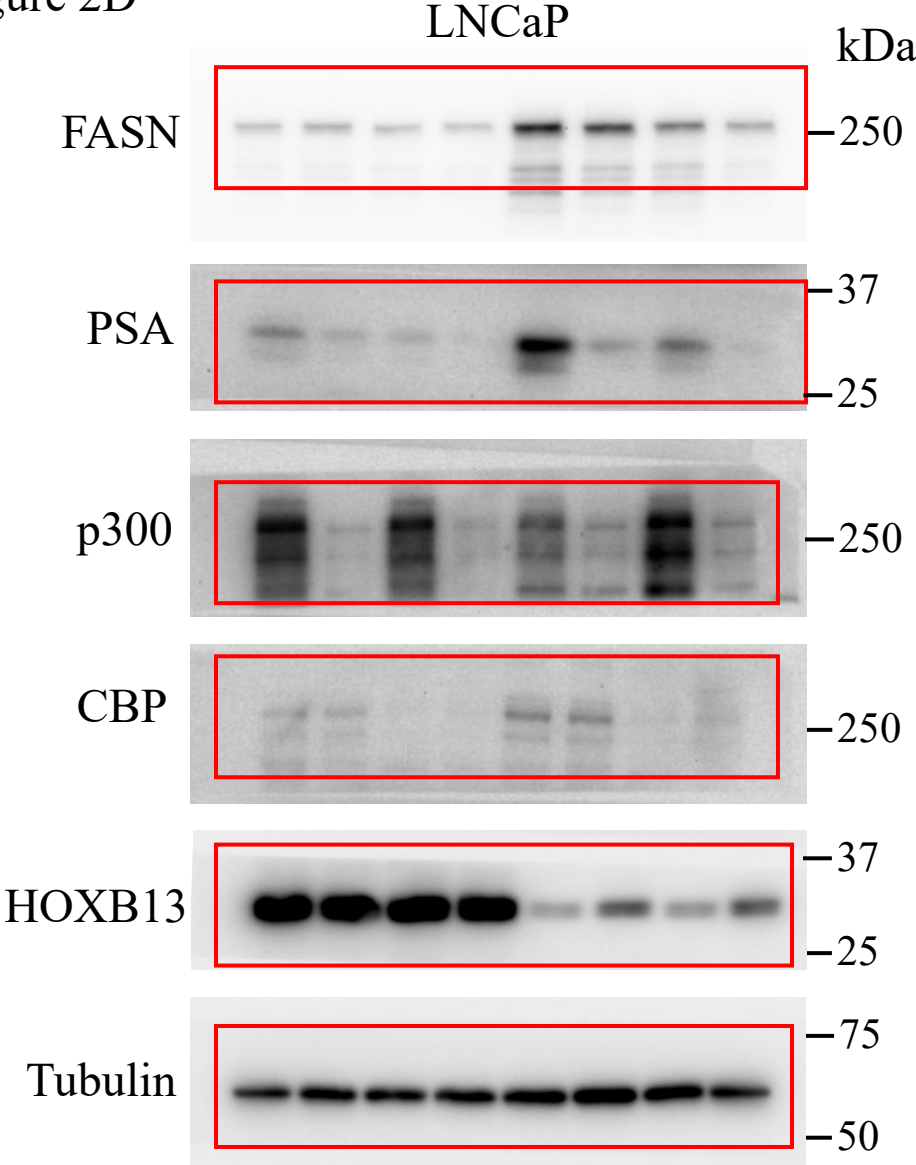

Figure 2E

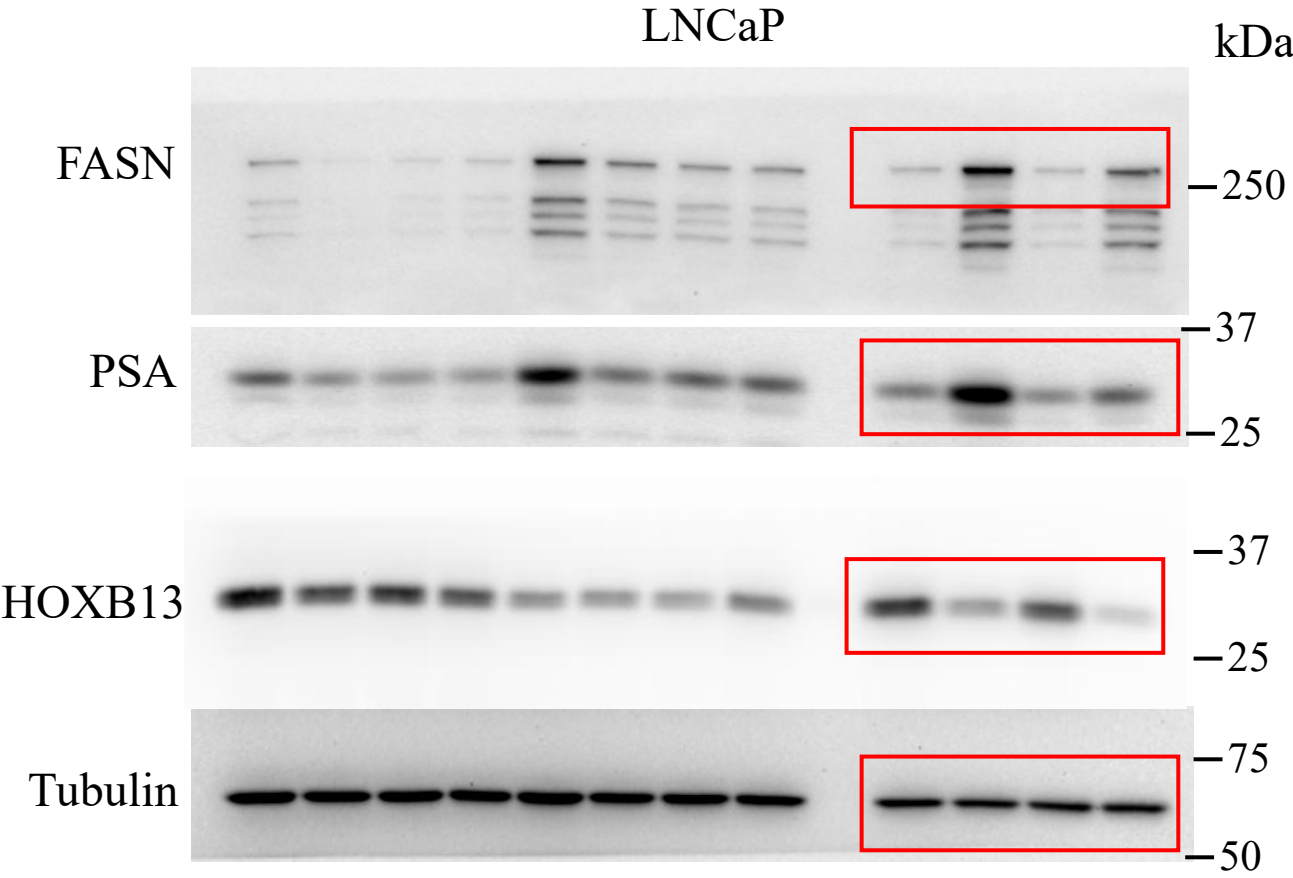

Supplemental Figure 1A

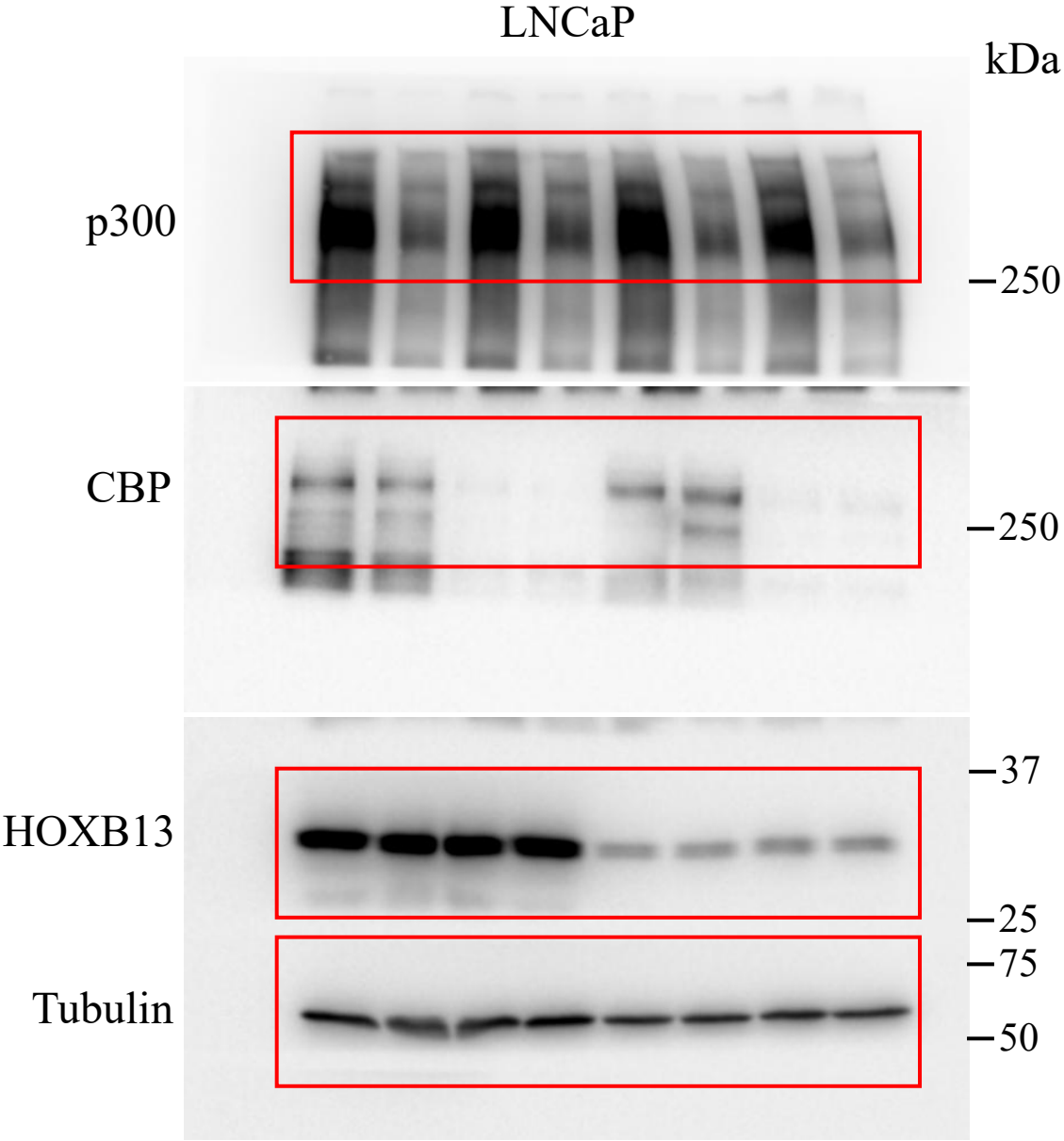

Supplemental Figure 2C

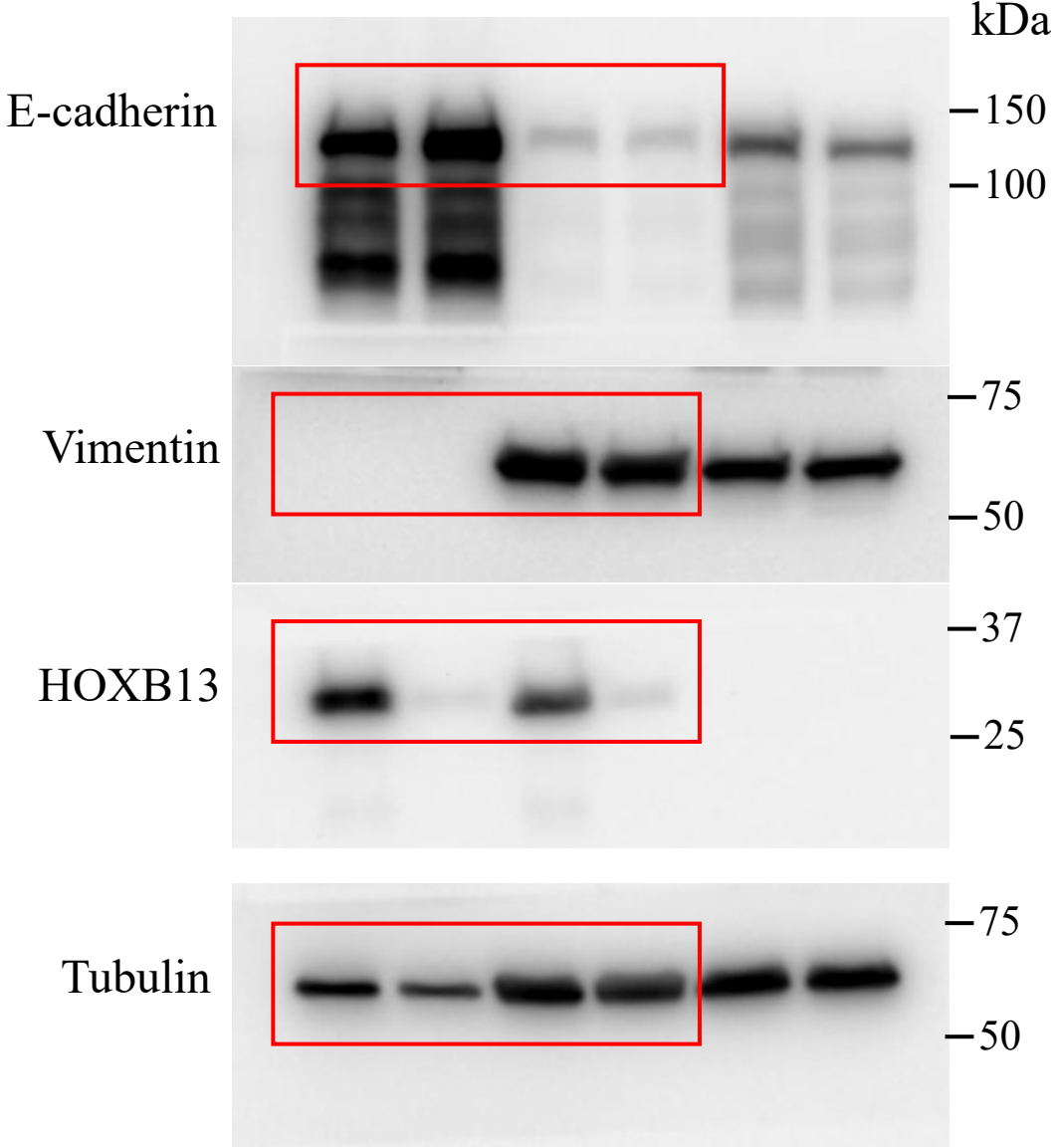

Supplemental Figure 3B

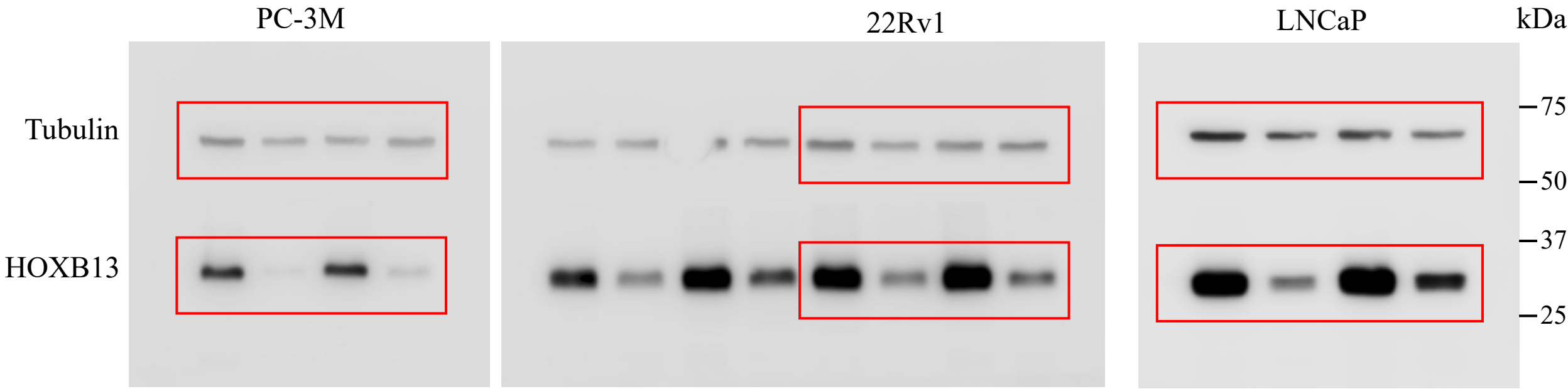

Supplement: Unedited blot and gel images [file jciinsight-10-195743-s112.pdf]
